# Trait–performance relationships of grassland plant species differ between common garden and field conditions

**DOI:** 10.1002/ece3.4818

**Published:** 2019-01-28

**Authors:** Eva Breitschwerdt, Ute Jandt, Helge Bruelheide

**Affiliations:** ^1^ Institute of Biology/Geobotany and Botanical Garden Martin Luther University Halle‐Wittenberg Halle Germany; ^2^ German Centre for Integrative Biodiversity Research (iDiv) Halle‐Jena‐Leipzig Leipzig Germany

**Keywords:** common garden experiment, land‐use, managed grassland, plant functional traits, plant performance, relative growth rates

## Abstract

The way functional traits affect growth of plant species may be highly context‐specific. We asked which combinations of trait values are advantageous under field conditions in managed grasslands as compared to conditions without competition and land‐use. In a two‐year field experiment, we recorded the performance of 93 species transplanted into German grassland communities differing in land‐use intensity and into a common garden, where species grew unaffected by land‐use under favorable conditions regarding soil, water, and space. The plants’ performance was characterized by two independent dimensions (relative growth rates (RGR) of height and leaf length vs. aboveground biomass and survival) that were differently related to the eight focal key traits in our study (leaf dry matter content (LDMC), specific leaf area (SLA), height, leaf anatomy, leaf persistence, leaf distribution, vegetative reproduction, and physical defense). We applied multivariate procrustes analyses to test for the correspondence of the optimal trait–performance relationships between field and common garden conditions. RGRs were species‐specific and species ranks of RGRs in the field, and the common garden were significantly correlated. Different traits explained the performance in the field and the common garden; for example, leaf anatomy traits explained species performance only in the field, whereas plant height was found to be only important in the common garden. The ability to reproduce vegetatively, having leaves that are summer‐persistent and with high leaf dry matter content (LDMC) were traits of major importance under both settings, albeit the magnitude of their influence differed slightly between the field and the common garden experiment. All optimal models included interactions between traits, pointing out the necessity to analyze traits in combination. The differences between field and common garden clearly demonstrate context dependency of trait‐based growth models, which results in limited transferability of favorable trait combinations between different environmental settings.

## 1 INTRODUCTION

Plant functional traits are connected with species differences in productivity and performance (Comas, Becker, Cruz, Byrne, & Dierig, 2013; Enquist et al., 2007; Poorter & Bongers, 2006). Moreover, it has been shown that traits strongly depend on the environment (Bruelheide et al., 2018; Díaz, Cabido, & Casanoves, 1998). Therefore, different combinations of traits may be advantageous under different environmental conditions. For example, under field conditions in managed grasslands other traits may be important as compared to conditions without land‐use and competition. However, it is still poorly understood how trait–performance relationships of different plant species vary under different environmental settings.

Differences in relative growth rates (RGRs) reflect species‐specific adaptations to abiotic factors such as climate, water, nutrient, and light availability as well as biotic factors such as competition, pathogens, or herbivory (Bultynck, Fiorani, & Lambers, 1999; Poorter, 1989). Stress‐tolerant species are considered to have low potential RGRs suitable for environments with low nutrient supply, whereas species with high potential RGRs are superior in highly productive habitats (Grime & Hunt, 1975; Lambers & Poorter, 1992). However, RGR of the same species varies with environment.

Under common garden conditions, species can be grown under favorable conditions regarding soil and water and growth is unaffected by mowing, grazing, or negative species interactions such as competition. RGRs under these conditions can be expected to be higher and approach the species’ potential growth rates, compared to natural field conditions.

Under controlled conditions, species RGRs are expected to be mainly correlated with leaf traits (Grime et al., 1997). High leaf dry matter content (LDMC) of plant species indicates low productive species, whereas high specific leaf area (SLA) is considered characteristic of competitive species (Suter & Edwards, 2013). For example, in a greenhouse experiment high potential growth rates were found to be correlated with high SLA (Hunt & Cornelissen, 1997; Poorter & Remkes, 1990), which, however, is not universally true, as was demonstrated for woody species (Böhnke & Bruelheide, 2013; Paine et al., 2015). This contrast between high LDMC and high SLA that distinguishes slow from fast‐growing species is known as the leaf economics spectrum (Wright et al., 2004).

In contrast, under realistic field conditions in managed grasslands abiotic and biotic factors can be expected to reduce species’ growth rates. Disturbances caused by land‐use have strong influences on species growth (Deng, Sweeney, & Shangguan, 2014; Herz et al., 2017). Furthermore, resources have to be shared with other species or defended against herbivores, which both can limit growth rates (Lind et al., 2013). However, plants were also found to increase growth rates to compensate for biomass loss from grazing (Zheng et al., 2014). Under realistic field conditions, other traits may be advantageous than in interaction‐free environments. For example, under high grazing pressure plants may grow smaller (Lienin & Kleyer, 2012) and/or have chemical and physical defense traits to avoid or reduce herbivory (Hanley, Lamont, Fairbanks, & Rafferty, 2007). Disturbances by trampling may benefit plants with clonal growth organs as they are able to invade much faster into disturbed areas (Bullock et al., 2001; Klimešová, Latzel, Bello, & Groenendael, 2008). Competition between plant species may also favor species with certain traits, for example, it has been reported that under competition for nutrients plants grow longer roots or under competition for light plants grow taller (Craine & Dybzinski, 2013). Roscher et al. (2011) found that a combination of monoculture biomass, plant growth rates, and resource‐use traits associated with nutrient and light acquisition best explained non‐legume species performance in a grassland biodiversity experiment.

In order to analyze the effects of different environmental settings on trait–performance relationships, we conducted two different experiments: A field experiment with impacts of land‐use and biotic interactions within the community and a common garden experiment where individual plants grew without land‐use and communities. For the field experiment, we established a large transplant experiment in mesic grasslands differing in land‐use intensity, management, and species composition in the three regions Schorfheide, Hainich, and Schwäbische Alb of Germany. A total of 2,592 individuals of 130 different grassland species new to the communities were planted into 54 grassland plots and monitored for growth over 2 years. Parallel to this field experiment, all 130 species were also grown in the common garden experiment at the Botanical Garden of the Martin Luther University Halle‐Wittenberg in Germany. In this study, we focused on the performance (biomass, survival and RGR) of 93 species out of the 130 planted species, because of mortality of some species during the two‐year observation time. To disentangle the correlations between performance and traits of plant species, we compared the performance of individuals grown under natural conditions, with the performance of the same species grown under common garden conditions, where species grew under favorable conditions concerning soil, water, and competition regimes. In this context, we tested two main hypotheses:

1. We expected that growth rates are highly species‐specific and that abiotic and biotic factors under field conditions reduce relative growth rates (RGRs), but result in similar overall patterns compared to RGRs in the common garden. In particular, we hypothesized that the species’ RGRs observed in the field experiment correspond to the RGRs when grown under common garden conditions.
2. Secondly, we hypothesized that the magnitude of growth and performance are correlated with different plant traits in field and common garden. In particular, we expected strong correlations with traits of the leaf economics spectrum (LES) (Wright et al., 2004) in the common garden experiment, whereas the traits vegetative reproduction and physical defense should be more relevant in the field experiment.

## 2 METHODS

### 2.1 Field experiment

Different grassland species were planted into managed grassland communities, making use of the network of experimental plots in the German Biodiversity Exploratories (Fischer et al., 2010). In each of the three study regions (Schwäbische Alb, South Germany; Hainich, Central Germany and Schorfheide, Northeast Germany), 18 grassland plots were selected, each six of them representing the three main land‐use types (meadow, pasture, and mown pasture). The plots differed in land‐use intensity, species richness, and functional diversity. Each plot was divided into eight subplots of 1 × 1 m which each were planted with six phytometers of six different species, selected from a total pool of 130 species. The six species planted in every subplot were selected specifically based on every plot's species composition, and thus differed among plots. Detailed information of the experimental design and the different planting scenarios is reported in Breitschwerdt, Jandt, and Bruelheide (2015). The experimental design resulted in different amounts of individuals per species across all plots. In total, we planted 2,592 individuals (3 Exploratories (= regions), 18 plots per Exploratory, 8 subplots per plot, 6 plant individuals per subplot).

After being planted in April 2012, the phytometers were monitored regularly for growth and survival in 2012 in April, May, July, August, and October and in 2013 in May, June/July, and September. At each date, we recorded height, aboveground plant projection area calculated from two diameters using the ellipse formula, length of leaves, and number of leaves. At the last monitoring date in September 2013, aboveground biomass of all surviving plants was harvested, dried, and weighed.

### 2.2 Common garden experiment

All 130 species used for transplantations in the field were also planted in a common garden experiment at the Botanical Garden of the Martin Luther University Halle‐Wittenberg in Germany in a threefold repetition in April 2012. In each of three gardening patches, rows of 12 individuals each with 0.25 cm distance to each other were established with one individual per plant species. All species were assigned randomly to their planting positions. The experiment was regularly weeded and watered. Snails and slugs were removed from the patches. Furthermore, we installed mollusk barriers consisting of metal sheets, buried 0.1 m belowground and extending 0.2 m aboveground to exclude slugs and snails. In addition, the metal was bent outward and lubricated with lemon‐based mollusk repellent (IRKA^®^ Schneckenabwehrpaste, Germany). In addition, we spread slug pellets (Neudorff Ferramol^®^ Schneckenkorn, Germany). Despite these exclosures, mollusks still caused damage on some plants. All phytometers were monitored for growth and survival in 2012 in April, May, June, and August and in 2013 in May and July/August. The same growth variables (height, aboveground plant projection area, leaf length, and number of leaves) were recorded as in the field experiment. Aboveground biomass was harvested at the end of the vegetation period in 2013.

### 2.3 Data analysis

Relative growth rates (RGR) were calculated for every individual plant according to formula (1) (Hoffmann & Poorter, 2002; Hunt & Cornelissen, 1997), where *M* is any growth variable and *t* is the time span in weeks between the two monitoring dates 1 and 2.

(1) $${\text{RGR}}_{i}=\frac{\ln\left(M_{2}\right)-\ln\left(M_{1}\right)}{t_{2}-t_{1}}$$

We calculated RGR mean values for all variables (height, plant projection area, leaf length, and number of leaves) per species. Therefore, we first aggregated the different time spans of the RGR of the both experiments per species and variable and then formed one RGR mean value per species and variable over all time spans (six time spans in the field and five in the common garden). Survival was calculated by taking the percentage of individuals per species that survived until the end of the experiments in relation to the amount of individuals per species planted at the start of the experiment. Total biomass at the end of the experiments in 2013 was aggregated to mean value per species and then log transformed to achieve normal distribution. As in both experiments (field and common garden), different species survived until the end, data on some of the 130 species had to be discarded, yielding to a total of 93 remaining species. The numbers of individuals that were included in the mean value calculations for each of the 93 species are shown in Supporting Information Table S2.

For the 93 species, we compiled a full trait matrix of eight traits (SLA, LDMC, height, leaf anatomy (succulent, scleromorphic, mesomorphic, hygromorphic, helomorphic, and hydromorphic), leaf persistence (in spring, summer or overwinter green or evergreen), leaf distribution (evenly spread leaves, rosettes, or semi‐rosettes), physical defense, and vegetative reproduction). Trait values were measured in 2011 (Breitschwerdt et al., 2015) and complemented from the databases LEDA (Kleyer et al., 2008), BIOPOP (Poschlod, Kleyer, Jackel, Dannemann, & Tackenberg, 2003), BIOLFLOR (Klotz, Kühn, & Durka, 2002), and Rothmaler (Jäger & Werner, 2001). The species‐mean values of all traits are provided in Supporting Information Table S1. As none of the 93 species had hydromorphic leaves or leaves that are persistent over winter, these trait states were excluded from calculations. Furthermore, we excluded leaf persistence evergreen and leaf distribution evenly spread leaves from analyses to avoid redundant information.

### 2.4 PCA and procrustes analyses

We performed principal component analyses (PCAs) with the vegan package of R (Oksanen et al., 2015). We based the calculations on all species‐mean performance variables (mean RGR of height, plant projection area, leaf length, and number of leaves, biomass and survival rates), and carried out PCAs separately for the field experiment and the common garden experiment.

These performance PCAs of field and common garden observations were compared to a PCA based on all species traits, using procrustes rotation (procrustes function in vegan). Applied to the pair of performance and trait PCAs, the procedure rotates and scales the PC scores of the second PCA to maximally fit those of the first target PCA, minimizing the sum of squared differences. To test whether different traits were relevant in the field and the common garden, we then searched for an optimized corresponding trait PCA that best explained performance, using a forward selection of traits, also including two‐way interaction of traits. This optimization procedure was carried out separately for the field and common garden PCA. We developed a stepwise forward selection by adding that trait or trait interaction to the trait PCA that resulted in the best correlation in a symmetric procrustes rotation between the performance and trait matrix. The correlation coefficient was obtained by the protest command of the procrustes analyses in vegan package of R (Oksanen et al., 2015), using 9,999 permutations. We run this forward selection until further addition of traits or trait interactions did no longer improve the procrustes correlation coefficient between each of the two performance PCAs and the corresponding trait PCA. We also considered forward selection of predictors using redundancy analyses (RDAs), but in comparison with the procrustes approach found RDA to be too greedy, resulting in much longer final trait lists. Furthermore, the automated forward procedure (ordistep or ordiR2step in vegan) could not be used, because the trait states of the same trait had to enter the model as a group (e.g., leaf anatomy, leaf persistence, and leaf distribution), also interactions with the different trait states had be handled as group and once discarded traits had be considered in subsequent steps. Therefore, we considered procrustes analyses the most appropriate way to select the best combination of predictor traits.

### 2.5 Univariate analyses

In addition, we employed ordinary linear regression models, relating the final traits to all performance variables of the field and the common garden experiment. Furthermore, the species’ ranks of each performance variable in both experiments (field and common garden) were compared using a Spearman correlation test.

## 3 RESULTS

All performance variables except RGR plant projection area showed a strong correlation between the mean values of the 93 study species obtained in the field compared to the common garden experiment according to Spearman's rank correlation coefficient (see Supporting Information Table S4). The best match was encountered for biomass production (*r*_s_ = 0.42), followed by RGR leaf length and survival (each 0.32), RGR height (0.28), and RGR number of leaves (0.22).

**Figure 1:**
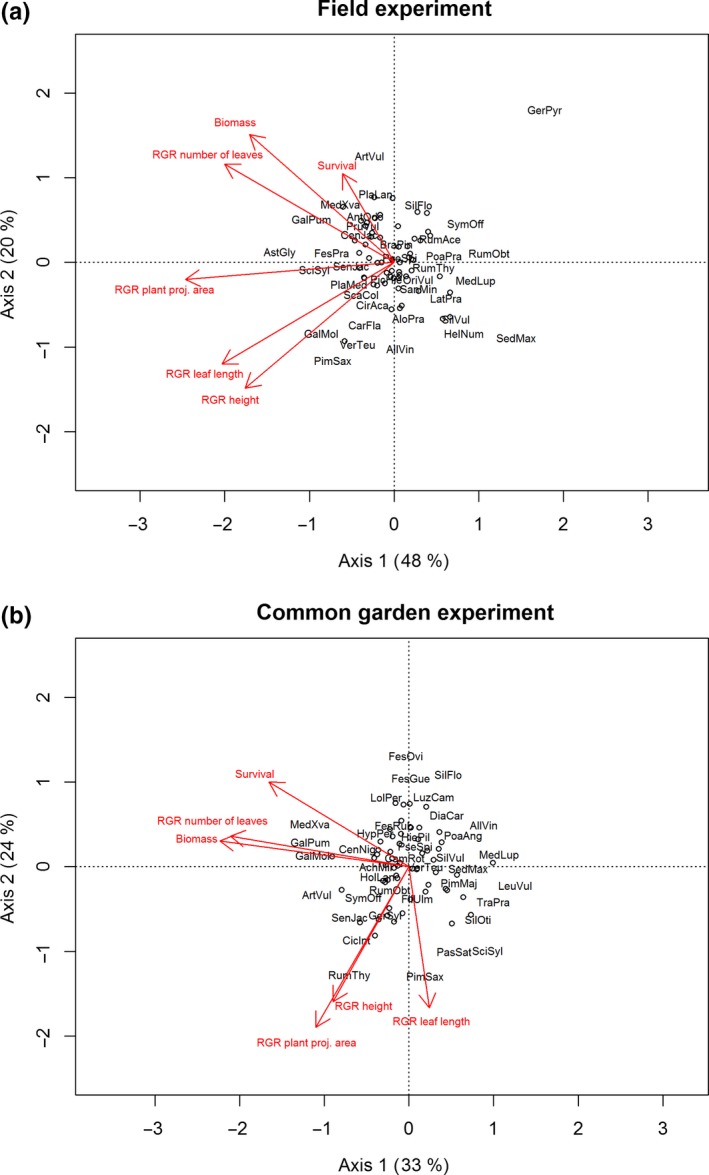
PCA of 93 species (abbreviations see Supporting information Table S1) based on mean relative growth rates (RGR) of height, plant projection area, leaf length, number of leaves, biomass, and survival (a) in the field experiment and (b) in the common garden experiment. Explained variance of axes is given in percentage. Eigenvalues of the first two PCA axes in (a) were 2.88 and 1.18 and in (b) 1.99 and 1.42

Principal components analyses (PCA) of all species based on mean relative growth rates of height, plant projection area, leaf length, number of leaves, biomass, and survival showed very similar relationships between performance variables in the field and in the common garden experiment (Figure 1a,b).

While in both PCAs, the first axis reflected relative growth rates, in the field they were mostly related to projection area and in the common garden to leaf number as well as to biomass. The second PCA axis both in the field and common garden was characterized by positive loadings of survival, biomass, and RGR of number of leaves and negative ones for increasing values of RGR of height, leaf length, and plant projection area (Figure 1a,b).

Species with lowest scores on the first PCA axis, and thus, highest performance, were *Astragalus glycyphyllos* (AstGly), *Galium pumilum* (GalPum), and *Scirpus sylvaticus* (SciSyl) in the field and *Medicago x varia* (MedXva), *Galium pumilum* (GalPum), and *Galium mollugo* (GalMol) the common garden (Figure 1a,b, for scores of all species see Supporting Information Table S3). Low scores on the second PCA axis, and thus high RGR in height, leaf number, or projection area, were found for *Pimpinella saxifraga* (PimSax), *Allium vineale* (AllVin), *Veronica teucrium* (VerTeu), and *Galium mollugo* (GalMol) in the field and for *Pimpinella saxifraga* (PimSax), *Rumex thyrsiflorus* (RumThy), and *Pastinaca sativa* (PasSat) in the common garden experiment (Figure 1a,b).

**Figure 2:**
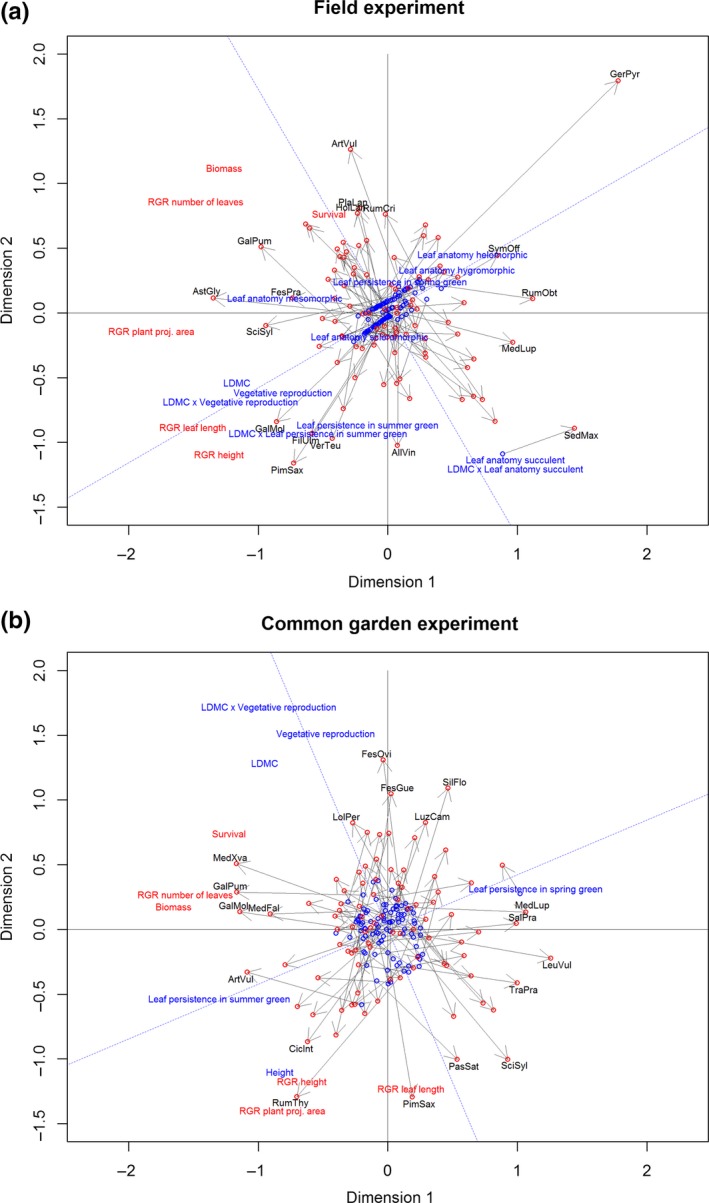
Procrustes analyses of PCAs: (a) PCA of traits in the field rotated to match PCA of performance in the field; (b) PCA traits in the common garden rotated to match PCA performance in the common garden. For the field experiment, the optimized remaining traits were as follows: LDMC, leaf anatomy (succulent, scleromorphic, mesomorphic, hygromorphic, and helomorphic), leaf persistence (persistent in spring and persistent in summer), vegetative reproduction, and the three interaction traits between LDMC with leaf anatomy succulent, persistence summer, and vegetative reproduction. For the common garden experiment, the optimized remaining traits were as follows: LDMC, height, leaf persistence (persistent in spring and persistent in summer), vegetative reproduction, and the interaction between LDMC and vegetative reproduction. Arrows show procrustes errors (longer arrows = higher errors) calculated by rotating species in 9,999 permutations and comparing species positions of two PCA until finding positions with least differences. For abbreviations of species names see Supporting Information Table S1. Only species with highest scores on axes (above the 95th percentile or below the 5th percentile) are shown

Comparing the performance PCAs of the field and the common garden experiment with procrustes analyses resulted in a correlation of 0.31 (*p* = 0.0003, Table 1). The PCA of all traits showed that the dimensions of 14 traits were not well captured by only one or two axes, which explained 17% and 13% of variation in trait values (Supporting Information Figure S1). Thus, we used the whole ordination of traits as predictor for performance. In both PCAs of performance in the field and in the common garden, the procrustes correlation with the PCA based on all traits was insignificant (Table 1), showing the necessity to eliminate uninformative traits. The optimization process of the trait PCA to explain performance in the field experiment resulted in 12 traits (LDMC, leaf anatomy (five categories), leaf persistence (two categories), vegetative reproduction and the three interaction traits LDMC with anatomy succulent, persistence summer, and vegetative reproduction, Figure 2a). The optimized trait PCA explaining common garden performance contained six traits (LDMC, height, leaf persistence (two categories), vegetative reproduction, and the interaction of LDMC with vegetative reproduction (Figure 2b)). The procrustes correlation coefficients between the performance PCAs for field or common garden data, and the corresponding trait PCA based on the optimized set of traits were 31% and 37%, respectively (*p* = 0.028 and 0.0001, Table 1). However, the reciprocal application of trait PCA optimized for the field performance PCA to the common garden performance PCA and vice versa resulted in insignificant correlations (Table 1).

**Table 1:** Results of procrustes analyses based on the principal component analyses (PCAs) of all species' performance variables (RGR of height, plant projection area, leaf length and number of leaves, biomass, and survival) and traits

	Correlation in a symmetric procrustes rotation	Significance
PCA performance field vs. PCA performance CG	0.3134	0.0003
PCA performance field vs. PCA all traits	0.144	0.2717
PCA performance CG vs. PCA all traits	0.185	0.083
PCA performance field vs. PCA traits optimized for field	0.3106	0.0028
PCA performance CG vs. PCA traits optimized for CG	0.3673	0.0001
PCA performance field vs. PCA traits optimized for CG	0.139	0.3011
PCA performance CG vs. PCA traits optimized for field	0.1683	0.1438

Note

Traits in the field experiment were LDMC, leaf anatomy (succulent, scleromorphic, mesomorphic, hygromorphic, and helomoprhic), leaf persistence (green in spring or summer), vegetative reproduction, and the three interaction traits between LDMC with leaf anatomy succulent, leaf persistence green in summer and vegetative reproduction. Traits in the common garden experiment were LDMC, height, leaf persistence (green in spring green or in summer), vegetative reproduction, and the interaction between LDMC and vegetative reproduction.

The trait‐wise analyses of all performance variables in separate linear regression models (Table 2) reflected the results of the procrustes rotations (Figure 2). For example, relative growth rates of leaf length and height based on field observations were positively correlated with LDMC, vegetative reproduction, and the interaction between the two (Figure 2a). In accordance with the linear models, relative growth rate of height was also correlated with persistence in summer and its interaction with LDMC, relative growth rate of plant projection area with leaf anatomy mesomorphic (Figure 2a).

**Table 2:** Correlations between optimal traits found for each experiment (field and common garden) and the respective performance variables (biomass, survival, RGR of height, plant projection area, leaf length, and number of leaves) of each experiment

Traits field	Performance variables					
	Biomass	Survival	RGR of height	RGR of plant projection area	RGR of leaf length	RGR of number of leaves
LDMC	−0.099	0.048	0.097	0.119	0.143	0.152
Leaf anatomy succulent	−0.356***	−0.171	−0.198	−0.182	−0.112	−0.285**
Leaf anatomy scleromorphic	−0.022	−0.100	0.112	−0.001	0.050	0.038
Leaf anatomy mesomorphic	0.170	0.206*	0.021	0.210*	0.086	0.173
Leaf anatomy hygromorphic	−0.136	0.205*	−0.028	−0.174	−0.033	−0.145
Leaf anatomy helomorphic	0.144	−0.053	−0.061	0.016	−0.065	−0.018
Leaf persistence in spring green	−0.012	−0.162	0.262*	−0.077	−0.020	−0.114
Leaf persistence in summer green	−0.095	−0.153	0.196	0.179	0.201	0.115
Vegetative reproduction	0.000	0.218*	0.110	0.279**	0.301**	0.133
LDMC × Leaf anatomy succulent	−0.356***	−0.171	−0.198	−0.182	−0.112	−0.285**
LDMC × Leaf persistence green in summer	−0.097	−0.105	0.206*	0.176	0.188	0.128
LDMC × Vegetative reproduction	−0.055	0.184	0.062	0.203	0.215*	0.179
**Traits common garden**						
LDMC	0.089	0.216*	−0.214*	−0.111	−0.096	0.292**
Height	0.348***	0.162	0.162	0.131	0.255*	−0.064
Leaf persistence in spring green	−0.185	0.057	−0.051	−0.097	−0.131	−0.256*
Leaf persistence in summer green	0.169	0.102	0.198	0.100	0.151	0.117
Vegetative reproduction	0.035	0.157	−0.274**	−0.044	−0.124	0.137
LDMC × Vegetative reproduction	0.117	0.264*	−0.330**	−0.097	−0.138	0.280**

Note

**Figure 3:**
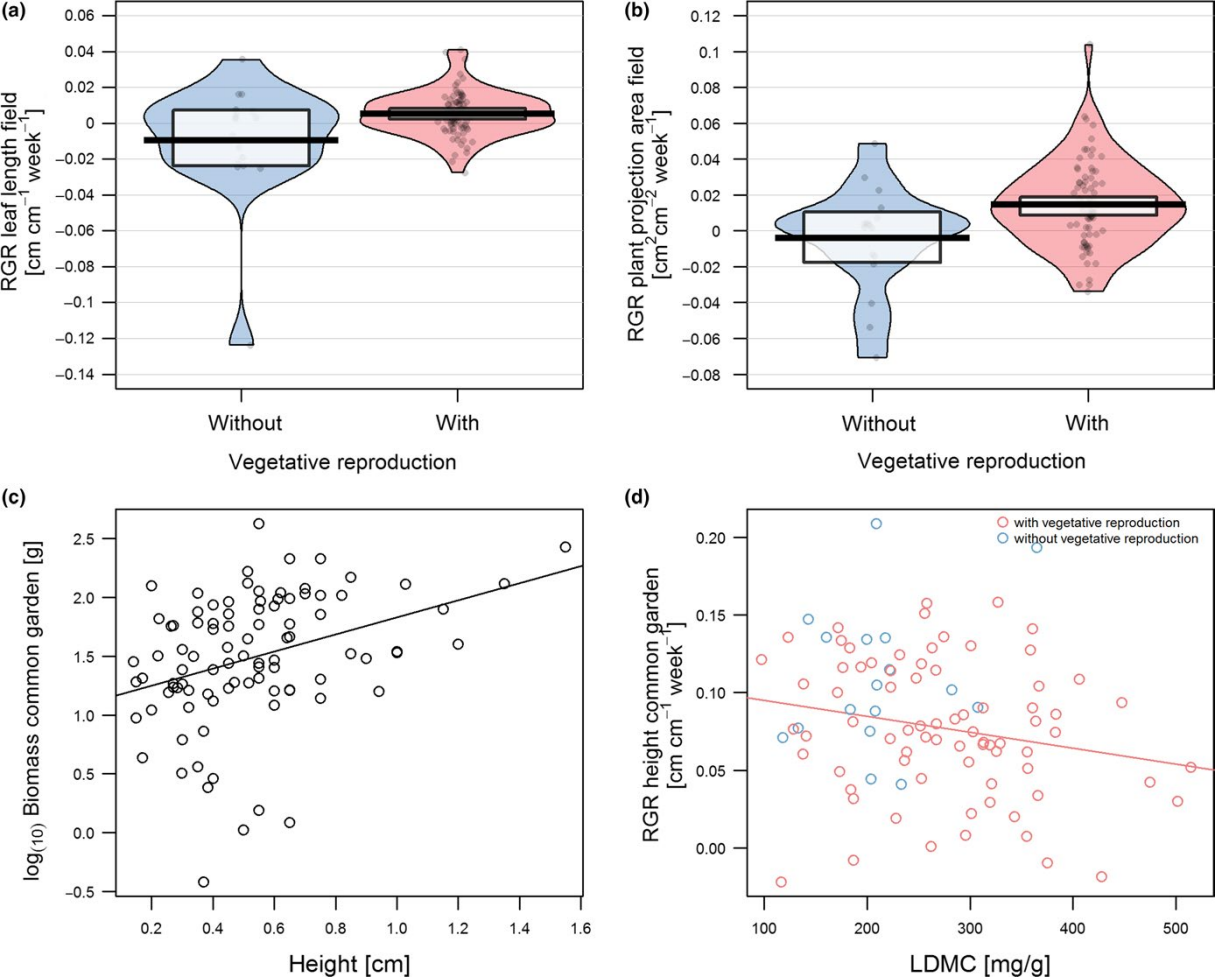
Correlations of (a) RGR leaf length in the field with vegetative reproduction, (b) RGR plant projection area in the field with the vegetative reproduction, (c) biomass in the common garden with the trait height, and (d) RGR height in the common garden with the interaction trait LDMC‐vegetative reproduction. Final traits were correlated in lm models with performance variables of field and common garden, respectively. The graphs show predictor variables with high correlation coefficients (for significance levels see Table 2)

Final traits were correlated in lm models in R with performance variables of field and common garden. Values are Pearson correlations coefficients. Significances are indicated with *. Significance levels are as following: from 0 to 0.001 = ***, from 0.001 to 0.01 = **, from 0.01 to 0.05 = *. Correlations in bold fonts are shown in Figure 3.

Differences among predictor traits between the procrustes analyses of the field and the common garden were LDMC, vegetative reproduction and their interaction which were positively correlated with relative growth rates of plant projection area, height, and leaf length in the field (Figure 2a), but negatively in the common garden (Figure 2b). In addition, there were positive correlations of survival with LDMC and the interaction between LDMC and vegetative reproduction in the common garden (Figure 2b) but not in the field (Figure 2a).

The comparison of the traits identified by the final procrustes models (Table 1), and the significant univariate relationships with performance variables (Table 2) reveals that multivariate relationships are not equally captured by univariate statistics. In the field experiment, RGRs of leaf length (Figure 3a) and plant projection area (Figure 3b) and survival were positively correlated with vegetative reproduction (Table 2). Furthermore, RGRs of plant projection area and survival were positively correlated with mesomorphic anatomy. RGR of height was positively correlated both with leaf persistence in spring and the interaction between LDMC and leaf persistence in summer (Table 2), showing that summer‐green species had overall higher mean RGRs of height with increasing LDMC in the field. Biomass and RGR number of leaves correlated negatively with succulent anatomy and the interaction of LDMC with succulent anatomy (Table 2).

In the common garden experiment, the finally selected traits displayed significant linear relationships with almost all performance variables except for RGR plant projection area (Table 2). Highest correlations were found between biomass and the plant height, showing that under unconstrained conditions biomass increased with potential plant height (Figure 3c). Similarly, RGR of leaf length was also positively correlated with plant height (Table 2). Negative correlations were found between RGR height and the interaction of LDMC and vegetative reproduction, showing that species with vegetative reproduction decreased in RGR height with increasing LDMC (Figure 3d). The single predictors LDMC and vegetative reproduction both had a negative impact on RGR height, while survival and RGR number of leaves increased with increasing LDMC and for species with vegetative reproduction (Table 2).

## 4 DISCUSSION

As expected, growth rates in the field were much smaller than under common garden conditions. Most aspects of growth as well as survival were species‐specific to some degree, as revealed by the significant rank correlations between field and common garden variables. These findings are consistent with the high congruence of the two performance PCAs based on field and common garden observations. Our first hypothesis stated that the species’ RGRs observed in the field experiment correspond to the species’ RGRs grown under common garden conditions, which was clearly confirmed by our findings. In this aspect, our study is in accordance with results of previous studies that describe only minor impacts of different environments on interspecific rankings (Al Haj Khaled, Duru, Theau, Plantureux, & Cruz, 2005; Garnier et al., 2001; Kazakou et al., 2014; Roche, Díaz‐Burlinson, & Gachet, 2004). For example, Meziane and Shipley (1999) described that ranks of net assimilation rates, which are related to relative growth rates, were not much affected by differences in light and nutrient supply. Performance data of species obtained from garden experiments are therefore good predictors for performance under field conditions.

Procrustes optimization identified different trait constellations that explained performance in both experiments, thus confirming our second hypothesis. The differences in the optimized trait combinations show that some species characteristics are only relevant under realistic management regimes and others under favorable garden conditions. Leaf anatomical traits were only important under field conditions, probably because they reflect the species’ photosynthetic capacity and are directly connected with growth rates and indirectly with recovery from defoliation by land‐use, which was irrelevant in the common garden. In contrast, potential height was only important in the common garden, where the plants could attain large sizes without being grazed or mown. There were even traits with opposing effects on growth. While the ability to reproduce vegetatively characterized slow‐growing species with respect to RGR leaf length in the common garden (e.g., *Festuca ovina*, *F. guestfalica,* and *Silene flos‐cuculi*), this trait was characteristic of fast‐growing species in the field (e.g., *Galium mollugo* and *Scirpus sylvaticus)*. In the field experiment, species with vegetative reproduction displayed increased RGR of height, leaf length, and plant projection area, while in the common garden experiment species with vegetative reproduction decreased in RGR. The different role of vegetative reproduction under different land‐use regimes has also been reported from studies in population ecology. Johansen, Wehn, and Hovstad (2016) reported that with decreasing grazing intensity clonal regeneration increased in importance of population growth rates of *Knautia arvensis*. These findings explain why the final sets of traits found for our two experiments were not interchangeable.

Nevertheless, there were also traits that played important roles under both fields and common garden conditions. LDMC and the interaction between LDMC and vegetative reproduction as well as leaf persistence were relevant under both conditions. The key role of these traits was also reported by Gross, Suding, and Lavorel (2007) who found that LDMC and lateral spread were suitable predictors of growth under different nutrient, shade, and clipping intensities. The importance of LDMC supported our expectation that LES traits are key predictors for performance. However, we found LDMC to be a better predictor for plant biomass production than SLA, as was pointed out also in previous studies (Kröber et al., 2015; Smart et al., 2017). More generally, leaf traits seem to be better predictors when based on mass rather than area (Lloyd, Bloomfield, Domingues, & Farquhar, 2013; Osnas, Lichstein, Reich, & Pacala, 2013). Overall, LES traits became only meaningful in combination with other traits. The final trait models in our study all included the ability to reproduce vegetatively, confirming previous findings that LES traits alone are poor predictors for plant growth (Paine et al., 2015). Similarly, LES traits were found to have a subordinate role in community assembly as response to land‐use. For example, Dirks, Dumbur, Lienin, Kleyer, and Grünzweig (2017) found that size and reproduction traits rather than leaf economic traits drove the composition of Mediterranean annual vegetation along a land‐use intensity gradient. This emphasizes the general importance of traits concerning clonal growth and vegetative reproduction for plant performance (Klimešová, Tackenberg, & Herben, 2016).

Furthermore, the interactions of LDMC with summer‐persistent leaves and succulent leaf anatomy were only relevant in the field. Thus, LDMC was not relevant for growth when species only had green leaves in spring, such as for *Allium vineale*. Similarly, a low LDMC did not translate into increased growth rate when the leaves were succulent, as for example in *Sedum maximum.* This combination of traits is characteristic for species with crassulacean acid metabolism (CAM), adapted to harsh and dry environments.

Against expectations, defense traits were not included in any final model, neither in the field nor in the common garden. These findings match the observations that community‐weighted physical defense traits did not respond to the land‐use gradient in the Biodiversity Exploratories (Plath & Bruelheide, unpublished results), pointing to a prevalence of plant strategies to tolerate grazing rather than to avoid the grazing impact in these grasslands. Instead, and unexpectedly, leaf anatomical traits turned out to be drivers of growth. Leaf anatomy traits are related to light absorption and photosynthetic rates, aspects also captured by LES traits. Comparing mesomorphic and scleromorphic leaves, the former display a higher membrane permeability and stromal conductance, leading to a higher photosynthetic capacity (Tomás et al., 2013). However, scleromorphic leaves were not advantageous in the field because species with mesomorphic leaves regrow more easily after mowing and grazing under real‐world land‐use conditions. As the field experiment was conducted in three different regions of Germany and contained grassland plots of different management regimes of grazing and mowing, future studies should aim at analyzing trait differences for particular land‐use types.

In conclusion, our study showed that species‐specific traits were capable to predict different dimensions of plant performance, characterized by relative growth rates and survival both under field and controlled common garden conditions. We found a prominent role of vegetative reproduction for plant performance, albeit with opposing effects under common garden and field conditions, and of LDMC. Importantly, additional traits and trait interactions modified plant performance under realistic field conditions. Thus, trait constellations and their interactions are not transferable across different environments. Overall, our study supports the necessity of including trait interactions into trait‐based plant growth models.

## CONFLICT OF INTEREST

The authors declare no competing financial interests.

## AUTHOR CONTRIBUTIONS

H.B. and U.J. developed the conceptual and methodological foundation of this study and designed the experiment. E.B. coordinated and collected field data. E.B. and H.B. conducted the statistical analyses, wrote the first draft of the manuscript, and prepared the figures.

## Data Availability

All data used in this paper is included in the Supporting Information (Tables S1–S3).
