# Supplementary material for: Trait–performance relationships of grassland plant species differ between common garden and field conditions
Source: Ecol Evol. 2019 Jan 28;9(4):1691–701. doi: 10.1002/ece3.4818 (PMC6392492; doi:10.1002/ece3.4818)
Supplement: Supplementary file 1 [file ECE3-9-1691-s001.docx]

**Supporting Information**

**Title:** Trait-performance relationships of grassland plant species differ between common garden and field conditions

**Authors:** Eva Breitschwerdt, Ute Jandt & Helge Bruelheide

**Ecology & Evolution**

**Table S1:** Species and traits. Trait values without missing values are for 93 species available. Trait values for each species are: SLA = specific leaf are, LDMC = leaf dry matter content, height, L.A. = leaf anatomy (suc = succulent, scler = scleromorphic, meso = mesomorphic, hygro = hygromorphic, helo = helomorphic), L.P. = leaf persistence (spri = green in spring, sum = green in summer, species with 0 in both categories have evergreen leaves), L.D. = leaf distribution (ros = rosettes, s-ros = semi-rosettes, species with 0 in both categories have evenly spread leaves), Phys. d. = physical defense, Veg. r. = vegetative reproduction.

| Species | Spec ID | SLA | LDMC | Height | L.A.suc | L.A.scler | L.A.meso | L.A.hygro | L.A.helo | L.P. spri | L.P. sum | L.D.ros | L.D.s-ros | Phys. d. | Veg. r. |
| --- | --- | --- | --- | --- | --- | --- | --- | --- | --- | --- | --- | --- | --- | --- | --- |
| Achillea millefolium | AchMil | 11.86 | 274.50 | 0.45 | 0 | 1 | 1 | 0 | 0 | 0 | 0 | 0 | 1 | 0 | 1 |
| Agrimonia eupatoria | AgrEup | 13.15 | 383.12 | 0.38 | 0 | 0 | 1 | 0 | 0 | 0 | 1 | 0 | 1 | 0 | 1 |
| Allium vineale | AllVin | 17.61 | 137.75 | 0.30 | 0 | 0 | 1 | 0 | 0 | 1 | 0 | 0 | 0 | 0 | 1 |
| Alopecurus pratensis | AloPra | 16.82 | 355.17 | 0.70 | 0 | 0 | 1 | 0 | 0 | 0 | 1 | 0 | 0 | 0 | 1 |
| Anthoxanthum odoratum | AntOdo | 21.08 | 262.12 | 0.22 | 0 | 0 | 1 | 0 | 0 | 0 | 0 | 0 | 0 | 0 | 1 |
| Arrhenatherum elatius | ArrEla | 23.92 | 313.30 | 1.03 | 0 | 0 | 1 | 0 | 0 | 0 | 1 | 0 | 0 | 0 | 1 |
| Artemisia vulgaris | ArtVul | 39.75 | 142.72 | 1.55 | 0 | 1 | 1 | 0 | 0 | 0 | 1 | 0 | 0 | 0 | 0 |
| Astragalus glycyphyllos | AstGly | 32.51 | 184.27 | 1.00 | 0 | 0 | 1 | 0 | 0 | 0 | 1 | 0 | 0 | 0 | 1 |
| Betonica officinalis | BetOff | 24.90 | 360.45 | 0.65 | 0 | 0 | 1 | 0 | 0 | 0 | 1 | 0 | 0 | 0 | 1 |
| Brachypodium pinnatum | BraPin | 15.04 | 474.77 | 0.55 | 0 | 1 | 1 | 0 | 0 | 0 | 1 | 0 | 0 | 0 | 1 |
| Briza media | BriMed | 29.56 | 320.63 | 0.35 | 0 | 1 | 1 | 0 | 0 | 0 | 0 | 0 | 1 | 0 | 1 |
| Bromus erectus | BroEre | 13.04 | 342.88 | 0.60 | 0 | 1 | 1 | 0 | 0 | 0 | 0 | 0 | 1 | 0 | 1 |
| Campanula rotundifolia | CamRot | 21.12 | 405.83 | 0.20 | 0 | 0 | 1 | 0 | 0 | 0 | 0 | 0 | 1 | 0 | 1 |
| Carex flacca | CarFla | 10.71 | 514.33 | 0.40 | 0 | 0 | 1 | 0 | 0 | 0 | 0 | 0 | 1 | 0 | 1 |
| Centaurea jacea | CenJac | 10.60 | 255.55 | 0.82 | 0 | 1 | 1 | 0 | 0 | 0 | 1 | 0 | 1 | 0 | 1 |
| Centaurea nigra | CenNig | 14.81 | 300.81 | 0.45 | 0 | 0 | 1 | 0 | 0 | 0 | 1 | 0 | 1 | 0 | 1 |
| Centaurea scabiosa | CenSca | 30.07 | 140.55 | 0.85 | 0 | 1 | 0 | 0 | 0 | 0 | 1 | 0 | 1 | 0 | 1 |
| Cichorium intybus | CicInt | 22.73 | 174.86 | 0.62 | 0 | 1 | 0 | 0 | 0 | 0 | 1 | 0 | 1 | 0 | 1 |
| Cirsium acaule | CirAca | 8.72 | 222.50 | 0.14 | 0 | 1 | 1 | 0 | 0 | 0 | 1 | 0 | 1 | 1 | 1 |
| Cynosurus cristatus | CynCri | 23.00 | 302.62 | 0.40 | 0 | 0 | 1 | 0 | 0 | 0 | 0 | 0 | 1 | 0 | 1 |
| Dianthus carthusianorum | DiaCar | 16.44 | 207.73 | 0.30 | 0 | 1 | 0 | 0 | 0 | 0 | 1 | 0 | 1 | 0 | 0 |
| Dipsacus fullonum | DipFul | 17.16 | 209.34 | 1.35 | 0 | 1 | 0 | 0 | 0 | 0 | 0 | 0 | 1 | 1 | 0 |
| Festuca arundinacea | FesAru | 20.27 | 382.86 | 1.20 | 0 | 1 | 1 | 0 | 0 | 0 | 1 | 0 | 0 | 0 | 1 |
| Festuca guestfalica | FesGue | 9.62 | 501.39 | 0.30 | 0 | 1 | 0 | 0 | 0 | 0 | 0 | 0 | 1 | 0 | 1 |
| Festuca ovina | FesOvi | 14.01 | 301.00 | 0.26 | 0 | 1 | 0 | 0 | 0 | 0 | 0 | 0 | 1 | 0 | 1 |
| Festuca pratensis | FesPra | 18.17 | 329.28 | 0.70 | 0 | 0 | 1 | 0 | 0 | 0 | 0 | 0 | 0 | 0 | 1 |
| Festuca rubra | FesRub | 10.21 | 365.90 | 0.51 | 0 | 0 | 1 | 0 | 0 | 0 | 0 | 0 | 1 | 0 | 1 |
| Filipendula ulmaria | FilUlm | 41.20 | 194.00 | 1.00 | 0 | 0 | 1 | 0 | 0 | 0 | 1 | 0 | 1 | 0 | 1 |
| Galium mollugo | GalMol | 29.62 | 231.33 | 0.51 | 0 | 0 | 1 | 0 | 0 | 0 | 1 | 0 | 0 | 0 | 1 |
| Galium pumilum | GalPum | 25.95 | 447.06 | 0.20 | 0 | 0 | 1 | 0 | 0 | 0 | 1 | 0 | 0 | 0 | 1 |
| Geranium pratense | GerPra | 14.47 | 266.40 | 0.40 | 0 | 0 | 1 | 1 | 0 | 0 | 1 | 0 | 1 | 0 | 1 |
| Geranium pyrenaicum | GerPyr | 24.65 | 183.62 | 0.47 | 0 | 0 | 1 | 0 | 0 | 0 | 0 | 0 | 1 | 0 | 0 |
| Geranium sylvaticum | GerSyl | 18.31 | 247.53 | 0.40 | 0 | 0 | 1 | 1 | 0 | 0 | 1 | 0 | 1 | 0 | 1 |
| Geum rivale | GeuRiv | 25.29 | 221.98 | 0.50 | 0 | 0 | 1 | 0 | 0 | 0 | 1 | 0 | 1 | 0 | 1 |
| Geum urbanum | GeuUrb | 14.15 | 312.29 | 0.75 | 0 | 0 | 1 | 1 | 0 | 0 | 0 | 0 | 1 | 0 | 1 |
| Helianthemum nummularium | HelNum | 15.19 | 252.92 | 0.15 | 0 | 1 | 0 | 0 | 0 | 0 | 0 | 0 | 0 | 0 | 1 |
| Helictotrichon pubescens | HelPub | 12.60 | 354.92 | 0.65 | 0 | 0 | 1 | 0 | 0 | 0 | 1 | 0 | 1 | 0 | 1 |
| Hieracium pilosella | HiePil | 14.18 | 295.25 | 0.17 | 0 | 0 | 1 | 0 | 0 | 0 | 0 | 1 | 0 | 0 | 1 |
| Holcus lanatus | HolLan | 28.19 | 267.25 | 0.65 | 0 | 0 | 1 | 0 | 0 | 0 | 0 | 0 | 0 | 0 | 1 |
| Hypericum perforatum | HypPer | 18.23 | 366.68 | 0.56 | 0 | 1 | 1 | 0 | 0 | 0 | 1 | 0 | 0 | 0 | 1 |
| Hypochaeris radicata | HypRad | 33.85 | 97.53 | 0.37 | 0 | 0 | 1 | 0 | 0 | 0 | 0 | 1 | 0 | 0 | 1 |
| Knautia arvensis | KnaArv | 21.52 | 176.14 | 0.55 | 0 | 0 | 1 | 0 | 0 | 0 | 1 | 0 | 1 | 0 | 1 |
| Koeleria pyramidata | KoePyr | 13.71 | 325.52 | 0.65 | 0 | 1 | 1 | 0 | 0 | 0 | 1 | 0 | 1 | 0 | 1 |
| Lathyrus pratensis | LatPra | 29.84 | 289.71 | 0.65 | 0 | 0 | 1 | 0 | 0 | 0 | 1 | 0 | 0 | 0 | 1 |
| Leontodon autumnalis | LeoAut | 26.07 | 171.37 | 0.30 | 0 | 0 | 1 | 0 | 0 | 0 | 0 | 1 | 0 | 0 | 1 |
| Leontodon hispidus | LeoHis | 14.97 | 285.42 | 0.28 | 0 | 0 | 1 | 0 | 0 | 0 | 1 | 1 | 0 | 0 | 1 |
| Leucanthemum vulgare | LeuVul | 22.67 | 138.10 | 0.50 | 0 | 1 | 1 | 0 | 0 | 0 | 0 | 0 | 1 | 0 | 1 |
| Lolium perenne | LolPer | 18.86 | 251.79 | 0.35 | 0 | 0 | 1 | 0 | 0 | 0 | 0 | 0 | 1 | 0 | 1 |
| Lotus corniculatus | LotCor | 20.03 | 252.42 | 0.22 | 0 | 0 | 1 | 0 | 0 | 0 | 1 | 0 | 0 | 0 | 1 |
| Luzula campestris | LuzCam | 20.01 | 227.85 | 0.15 | 0 | 1 | 1 | 0 | 0 | 0 | 1 | 0 | 1 | 0 | 1 |
| Medicago falcata | MedFal | 20.67 | 360.59 | 0.35 | 0 | 1 | 1 | 0 | 0 | 0 | 1 | 0 | 0 | 0 | 1 |
| Medicago lupulina | MedLup | 24.20 | 319.07 | 0.37 | 0 | 0 | 1 | 0 | 0 | 0 | 0 | 0 | 0 | 0 | 1 |
| Medicago x varia | MedXva | 24.56 | 239.90 | 0.55 | 0 | 0 | 1 | 0 | 0 | 0 | 0 | 0 | 0 | 0 | 1 |
| Origanum vulgare | OriVul | 13.24 | 358.58 | 0.40 | 0 | 1 | 1 | 0 | 0 | 0 | 1 | 0 | 0 | 0 | 1 |
| Pastinaca sativa | PasSat | 18.12 | 221.48 | 0.94 | 0 | 1 | 1 | 0 | 0 | 0 | 1 | 0 | 1 | 0 | 0 |
| Phleum phleoides | PhlPhl | 17.90 | 319.28 | 0.45 | 0 | 1 | 0 | 0 | 0 | 0 | 0 | 0 | 1 | 0 | 1 |
| Picris hieracioides | PicHie | 21.00 | 327.09 | 0.61 | 0 | 1 | 1 | 0 | 0 | 0 | 1 | 0 | 1 | 0 | 1 |
| Pimpinella major | PimMaj | 17.93 | 233.00 | 0.60 | 0 | 0 | 1 | 0 | 0 | 0 | 1 | 0 | 1 | 0 | 0 |
| Pimpinella saxifraga | PimSax | 12.00 | 364.91 | 0.32 | 0 | 1 | 1 | 0 | 0 | 0 | 1 | 0 | 1 | 0 | 0 |
| Plantago lanceolata | PlaLan | 17.61 | 204.11 | 0.30 | 0 | 1 | 1 | 0 | 0 | 0 | 1 | 1 | 0 | 0 | 1 |
| Plantago media | PlaMed | 15.85 | 183.22 | 0.27 | 0 | 1 | 1 | 0 | 0 | 0 | 1 | 1 | 0 | 0 | 1 |
| Poa angustifolia | PoaAng | 13.37 | 427.66 | 0.60 | 0 | 1 | 1 | 0 | 0 | 0 | 0 | 0 | 1 | 0 | 1 |
| Poa pratensis | PoaPra | 12.04 | 374.79 | 0.55 | 0 | 0 | 1 | 0 | 0 | 0 | 0 | 0 | 0 | 0 | 1 |
| Potentilla argentea | PotArg | 19.10 | 281.85 | 0.34 | 0 | 1 | 0 | 0 | 0 | 0 | 1 | 0 | 0 | 0 | 0 |
| Prunella vulgaris | PruVul | 23.29 | 222.56 | 0.17 | 0 | 0 | 1 | 0 | 0 | 0 | 0 | 0 | 0 | 0 | 1 |
| Pseudolysimachion spicatum | PseSpi | 41.11 | 128.24 | 0.27 | 0 | 1 | 0 | 0 | 0 | 0 | 0 | 0 | 0 | 0 | 1 |
| Ranunculus acris | RanAcr | 14.50 | 256.95 | 0.75 | 0 | 0 | 1 | 0 | 0 | 0 | 1 | 0 | 1 | 0 | 1 |
| Ranunculus repens | RanRep | 20.58 | 186.01 | 0.27 | 0 | 0 | 0 | 1 | 1 | 0 | 0 | 0 | 1 | 0 | 1 |
| Rumex acetosa | RumAce | 23.10 | 116.20 | 0.52 | 0 | 0 | 1 | 0 | 0 | 0 | 0 | 0 | 1 | 0 | 1 |
| Rumex crispus | RumCri | 15.36 | 199.38 | 0.90 | 0 | 0 | 1 | 0 | 1 | 0 | 0 | 0 | 1 | 0 | 0 |
| Rumex obtusifolius | RumObt | 23.98 | 160.04 | 0.85 | 0 | 0 | 1 | 1 | 0 | 0 | 0 | 0 | 1 | 0 | 0 |
| Rumex thyrsiflorus | RumThy | 18.36 | 208.54 | 0.75 | 0 | 0 | 1 | 0 | 0 | 0 | 0 | 0 | 1 | 0 | 0 |
| Salvia pratensis | SalPra | 20.38 | 186.37 | 0.45 | 0 | 1 | 1 | 0 | 0 | 0 | 1 | 0 | 1 | 0 | 1 |
| Sanguisorba minor | SanMin | 13.63 | 307.29 | 0.32 | 0 | 1 | 1 | 0 | 0 | 0 | 0 | 0 | 1 | 0 | 0 |
| Saponaria officinalis | SapOff | 20.09 | 363.46 | 0.55 | 0 | 0 | 1 | 0 | 0 | 0 | 1 | 0 | 0 | 0 | 1 |
| Scabiosa columbaria | ScaCol | 16.00 | 217.36 | 0.45 | 0 | 1 | 1 | 0 | 0 | 0 | 0 | 0 | 1 | 0 | 0 |
| Scirpus sylvaticus | SciSyl | 21.60 | 267.20 | 0.65 | 0 | 0 | 1 | 0 | 1 | 0 | 0 | 0 | 1 | 0 | 1 |
| Sedum maximum | SedMax | 17.74 | 172.98 | 0.55 | 1 | 0 | 0 | 0 | 0 | 0 | 1 | 0 | 0 | 0 | 1 |
| Senecio jacobaea | SenJac | 13.40 | 258.00 | 0.51 | 0 | 0 | 1 | 0 | 0 | 0 | 0 | 0 | 1 | 0 | 1 |
| Silaum silaus | SilSil | 13.93 | 293.66 | 0.65 | 0 | 1 | 1 | 0 | 0 | 0 | 1 | 0 | 1 | 0 | 1 |
| Silene dioica | SilDio | 37.11 | 133.14 | 0.60 | 0 | 0 | 1 | 0 | 0 | 0 | 0 | 0 | 1 | 0 | 0 |
| Silene flos-cuculi | SilFlo | 20.07 | 186.34 | 0.55 | 0 | 0 | 1 | 0 | 0 | 0 | 0 | 0 | 1 | 0 | 1 |
| Silene latifolia | SilLat | 19.93 | 170.90 | 0.64 | 0 | 0 | 1 | 0 | 0 | 0 | 0 | 0 | 0 | 0 | 1 |
| Silene otites | SilOti | 15.41 | 263.07 | 0.40 | 0 | 1 | 0 | 0 | 0 | 0 | 0 | 0 | 1 | 0 | 1 |
| Silene vulgaris | SilVul | 22.70 | 202.72 | 0.35 | 0 | 1 | 1 | 0 | 0 | 0 | 0 | 0 | 0 | 0 | 0 |
| Symphytum officinale | SymOff | 16.73 | 117.82 | 0.75 | 0 | 0 | 0 | 1 | 1 | 0 | 1 | 0 | 1 | 1 | 0 |
| Thymus pulegioides | ThyPul | 17.05 | 355.82 | 0.25 | 0 | 1 | 0 | 0 | 0 | 0 | 0 | 0 | 0 | 0 | 1 |
| Tragopogon pratensis | TraPra | 34.42 | 203.53 | 0.38 | 0 | 0 | 1 | 0 | 0 | 0 | 1 | 0 | 1 | 0 | 0 |
| Trisetum flavescens | TriFla | 25.05 | 312.49 | 0.55 | 0 | 0 | 1 | 0 | 0 | 0 | 0 | 0 | 0 | 0 | 1 |
| Valeriana officinalis | ValOff | 30.56 | 122.96 | 1.15 | 0 | 0 | 0 | 1 | 1 | 0 | 1 | 0 | 1 | 0 | 1 |
| Veronica teucrium | VerTeu | 20.34 | 238.47 | 0.60 | 0 | 1 | 1 | 0 | 0 | 0 | 1 | 0 | 0 | 0 | 1 |
| Vicia cracca | VicCra | 20.81 | 298.67 | 0.75 | 0 | 0 | 1 | 0 | 0 | 0 | 1 | 0 | 0 | 0 | 1 |
| Vicia sepium | VicSep | 23.41 | 236.04 | 0.45 | 0 | 0 | 1 | 0 | 0 | 0 | 0 | 0 | 0 | 0 | 1 |

**Table S2**: The 93 species and number of replicates per species (n) used in aggregation to mean values of relative growth rates (RGR) of height, plant projection area, leaf length and number of leaves obtained from field experiment (Field) and common garden experiment (CG). Furthermore the amount of individuals (ind.) of each species planted, alive at end of experiment and obtained biomass in both experiments. Abbreviations of species names are used in figures of PCA.

| **no.** | **Species** | **Abbr.** | **n ind. planted** | | **n ind. Survival** | | **n ind. Biomass** | | **n mean RGR height** | | **n mean RGR plant proj. area** | | **n mean RGR leaf length** | | **n mean RGR no. of leaves** | |
| --- | --- | --- | --- | --- | --- | --- | --- | --- | --- | --- | --- | --- | --- | --- | --- | --- |
|  |  |  | **Field** | **CG** | **Field** | **CG** | **Field** | **CG** | **Field** | **CG** | **Field** | **CG** | **Field** | **CG** | **Field** | **CG** |
| 1 | Achillea millefolium | AchMil | 50 | 3 | 36 | 3 | 36 | 3 | 230 | 15 | 230 | 15 | 229 | 15 | 229 | 15 |
| 2 | Agrimonia eupatoria | AgrEup | 6 | 3 | 3 | 3 | 4 | 3 | 28 | 15 | 28 | 15 | 27 | 15 | 27 | 15 |
| 3 | Allium vineale | AllVin | 16 | 3 | 3 | 3 | 1 | 3 | 12 | 9 | 12 | 9 | 10 | 10 | 10 | 9 |
| 4 | Alopecurus pratensis | AloPra | 30 | 3 | 5 | 3 | 5 | 3 | 81 | 13 | 81 | 13 | 81 | 15 | 81 | 15 |
| 5 | Anthoxanthum odoratum | AntOdo | 56 | 3 | 46 | 3 | 46 | 3 | 285 | 9 | 285 | 15 | 285 | 15 | 285 | 13 |
| 6 | Arrhenatherum elatius | ArrEla | 46 | 3 | 15 | 3 | 15 | 3 | 137 | 13 | 137 | 15 | 137 | 15 | 137 | 15 |
| 7 | Artemisia vulgaris | ArtVul | 12 | 3 | 5 | 3 | 5 | 3 | 46 | 13 | 46 | 13 | 40 | 13 | 40 | 13 |
| 8 | Astragalus glycyphyllos | AstGly | 12 | 3 | 4 | 3 | 4 | 3 | 32 | 12 | 32 | 12 | 32 | 12 | 32 | 12 |
| 9 | Betonica officinalis | BetOff | 14 | 3 | 6 | 3 | 6 | 3 | 58 | 15 | 58 | 15 | 58 | 15 | 58 | 15 |
| 10 | Brachypodium pinnatum | BraPin | 6 | 3 | 4 | 3 | 4 | 3 | 26 | 13 | 26 | 13 | 26 | 13 | 26 | 15 |
| 11 | Briza media | BriMed | 60 | 3 | 37 | 3 | 37 | 3 | 263 | 13 | 263 | 15 | 263 | 15 | 263 | 15 |
| 12 | Bromus erectus | BroEre | 12 | 3 | 8 | 3 | 8 | 3 | 55 | 9 | 55 | 13 | 55 | 15 | 55 | 13 |
| 13 | Campanula rotundifolia | CamRot | 4 | 3 | 3 | 3 | 3 | 3 | 18 | 15 | 18 | 15 | 18 | 15 | 18 | 15 |
| 14 | Carex flacca | CarFla | 8 | 3 | 2 | 2 | 2 | 2 | 16 | 11 | 16 | 11 | 16 | 11 | 16 | 11 |
| 15 | Centaurea jacea | CenJac | 42 | 3 | 25 | 3 | 25 | 3 | 186 | 13 | 186 | 15 | 186 | 15 | 186 | 15 |
| 16 | Centaurea nigra | CenNig | 18 | 3 | 9 | 3 | 9 | 3 | 68 | 13 | 68 | 13 | 66 | 13 | 66 | 13 |
| 17 | Centaurea scabiosa | CenSca | 28 | 3 | 14 | 2 | 14 | 2 | 109 | 13 | 109 | 11 | 107 | 13 | 107 | 13 |
| 18 | Cichorium intybus | CicInt | 50 | 3 | 20 | 2 | 20 | 2 | 181 | 11 | 181 | 11 | 181 | 11 | 179 | 11 |
| 19 | Cirsium acaule | CirAca | 8 | 3 | 4 | 3 | 4 | 3 | 31 | 15 | 31 | 15 | 31 | 15 | 31 | 15 |
| 20 | Cynosurus cristatus | CynCri | 8 | 3 | 6 | 3 | 6 | 3 | 43 | 13 | 43 | 11 | 43 | 15 | 43 | 15 |
| 21 | Dianthus carthusianorum | DiaCar | 36 | 3 | 13 | 3 | 13 | 3 | 112 | 15 | 112 | 13 | 112 | 15 | 112 | 15 |
| 22 | Dipsacus fullonum | DipFul | 98 | 3 | 21 | 3 | 30 | 3 | 393 | 15 | 393 | 15 | 386 | 15 | 386 | 15 |
| 23 | Festuca arundinacea | FesAru | 8 | 3 | 4 | 3 | 4 | 3 | 38 | 13 | 38 | 11 | 36 | 15 | 36 | 15 |
| 24 | Festuca guestfalica | FesGue | 2 | 3 | 2 | 3 | 2 | 3 | 12 | 13 | 12 | 15 | 12 | 15 | 10 | 15 |
| 25 | Festuca ovina | FesOvi | 14 | 3 | 10 | 3 | 10 | 3 | 67 | 13 | 67 | 13 | 67 | 13 | 56 | 15 |
| 26 | Festuca pratensis | FesPra | 24 | 3 | 17 | 3 | 16 | 3 | 115 | 15 | 115 | 13 | 115 | 15 | 115 | 15 |
| 27 | Festuca rubra | FesRub | 16 | 3 | 11 | 3 | 11 | 3 | 69 | 11 | 69 | 15 | 68 | 15 | 69 | 15 |
| 28 | Filipendula ulmaria | FilUlm | 10 | 3 | 6 | 3 | 6 | 3 | 37 | 15 | 37 | 15 | 37 | 15 | 37 | 11 |
| 29 | Galium mollugo | GalMol | 10 | 3 | 1 | 3 | 1 | 3 | 13 | 13 | 13 | 15 | 13 | 13 | 7 | 15 |
| 30 | Galium pumilum | GalPum | 10 | 3 | 3 | 3 | 3 | 3 | 23 | 15 | 23 | 15 | 23 | 13 | 17 | 15 |
| 31 | Geranium pratense | GerPra | 2 | 3 | 2 | 3 | 2 | 3 | 12 | 15 | 12 | 15 | 12 | 15 | 12 | 15 |
| 32 | Geranium pyrenaicum | GerPyr | 2 | 3 | 1 | 1 | 1 | 1 | 5 | 12 | 5 | 12 | 5 | 12 | 5 | 12 |
| 33 | Geranium sylvaticum | GerSyl | 2 | 3 | 2 | 3 | 2 | 3 | 12 | 15 | 12 | 15 | 12 | 15 | 12 | 15 |
| 34 | Geum rivale | GeuRiv | 12 | 3 | 8 | 3 | 8 | 3 | 52 | 15 | 52 | 11 | 52 | 15 | 52 | 15 |
| 35 | Geum urbanum | GeuUrb | 12 | 3 | 10 | 3 | 10 | 3 | 66 | 15 | 66 | 15 | 66 | 13 | 66 | 13 |
| 36 | Helianthemum nummularium | HelNum | 6 | 3 | 2 | 1 | 2 | 1 | 18 | 9 | 18 | 9 | 18 | 9 | 18 | 9 |
| 37 | Helictotrichon pubescens | HelPub | 8 | 3 | 4 | 3 | 4 | 3 | 31 | 9 | 31 | 13 | 31 | 13 | 31 | 13 |
| 38 | Hieracium pilosella | HiePil | 26 | 3 | 13 | 2 | 13 | 2 | 99 | 7 | 99 | 11 | 99 | 10 | 99 | 10 |
| 39 | Holcus lanatus | HolLan | 82 | 3 | 68 | 3 | 68 | 3 | 423 | 13 | 423 | 15 | 423 | 15 | 423 | 15 |
| 40 | Hypericum perforatum | HypPer | 14 | 3 | 9 | 3 | 9 | 3 | 60 | 15 | 60 | 15 | 60 | 15 | 60 | 13 |
| 41 | Hypochaeris radicata | HypRad | 4 | 3 | 4 | 1 | 4 | 1 | 24 | 11 | 24 | 11 | 24 | 11 | 24 | 11 |
| 42 | Knautia arvensis | KnaArv | 14 | 3 | 7 | 3 | 7 | 3 | 61 | 13 | 59 | 15 | 59 | 15 | 59 | 13 |
| 43 | Koeleria pyramidata | KoePyr | 4 | 3 | 4 | 3 | 4 | 3 | 24 | 15 | 24 | 11 | 24 | 15 | 24 | 15 |
| 44 | Lathyrus pratensis | LatPra | 10 | 3 | 3 | 3 | 3 | 3 | 34 | 11 | 34 | 10 | 34 | 9 | 34 | 11 |
| 45 | Leontodon autumnalis | LeoAut | 16 | 3 | 8 | 1 | 8 | 1 | 62 | 10 | 62 | 10 | 62 | 9 | 62 | 9 |
| 46 | Leontodon hispidus | LeoHis | 12 | 3 | 6 | 2 | 6 | 2 | 48 | 13 | 48 | 13 | 48 | 13 | 48 | 13 |
| 47 | Leucanthemum vulgare | LeuVul | 6 | 3 | 3 | 1 | 3 | 1 | 28 | 7 | 28 | 9 | 28 | 9 | 28 | 9 |
| 48 | Lolium perenne | LolPer | 28 | 3 | 12 | 3 | 12 | 3 | 119 | 15 | 119 | 15 | 119 | 15 | 119 | 15 |
| 49 | Lotus corniculatus | LotCor | 72 | 3 | 40 | 3 | 40 | 3 | 301 | 15 | 301 | 15 | 299 | 15 | 272 | 15 |
| 50 | Luzula campestris | LuzCam | 30 | 3 | 16 | 3 | 16 | 3 | 129 | 9 | 129 | 11 | 129 | 15 | 129 | 15 |
| 51 | Medicago falcata | MedFal | 4 | 3 | 2 | 3 | 2 | 3 | 15 | 15 | 15 | 15 | 15 | 15 | 13 | 13 |
| 52 | Medicago lupulina | MedLup | 12 | 3 | 1 | 1 | 1 | 1 | 26 | 10 | 26 | 6 | 26 | 9 | 20 | 9 |
| 53 | Medicago x varia | MedXva | 6 | 3 | 5 | 3 | 5 | 3 | 30 | 15 | 30 | 15 | 30 | 15 | 26 | 15 |
| 54 | Origanum vulgare | OriVul | 10 | 3 | 6 | 3 | 6 | 3 | 38 | 15 | 38 | 15 | 38 | 15 | 38 | 15 |
| 55 | Pastinaca sativa | PasSat | 28 | 3 | 7 | 1 | 7 | 1 | 86 | 11 | 86 | 11 | 79 | 11 | 79 | 11 |
| 56 | Phleum phleoides | PhlPhl | 10 | 3 | 4 | 3 | 4 | 3 | 40 | 9 | 40 | 15 | 40 | 15 | 40 | 15 |
| 57 | Picris hieracioides | PicHie | 20 | 3 | 7 | 2 | 9 | 2 | 83 | 13 | 83 | 13 | 82 | 13 | 82 | 13 |
| 58 | Pimpinella major | PimMaj | 6 | 3 | 4 | 2 | 4 | 2 | 25 | 6 | 25 | 8 | 25 | 8 | 25 | 8 |
| 59 | Pimpinella saxifraga | PimSax | 8 | 3 | 2 | 2 | 2 | 2 | 24 | 7 | 24 | 9 | 24 | 8 | 24 | 7 |
| 60 | Plantago lanceolata | PlaLan | 12 | 3 | 7 | 2 | 7 | 2 | 50 | 11 | 50 | 13 | 48 | 12 | 48 | 12 |
| 61 | Plantago media | PlaMed | 6 | 3 | 4 | 3 | 4 | 3 | 31 | 13 | 31 | 15 | 31 | 13 | 31 | 15 |
| 62 | Poa angustifolia | PoaAng | 4 | 3 | 1 | 3 | 1 | 3 | 6 | 9 | 6 | 15 | 6 | 15 | 6 | 15 |
| 63 | Poa pratensis | PoaPra | 4 | 3 | 3 | 3 | 3 | 3 | 19 | 9 | 19 | 13 | 19 | 15 | 19 | 15 |
| 64 | Potentilla argentea | PotArg | 74 | 3 | 29 | 3 | 29 | 3 | 313 | 15 | 313 | 15 | 311 | 15 | 311 | 15 |
| 65 | Prunella vulgaris | PruVul | 56 | 3 | 38 | 3 | 38 | 3 | 246 | 15 | 246 | 15 | 246 | 15 | 246 | 15 |
| 66 | Pseudolysimachion spicatum | PseSpi | 22 | 3 | 11 | 3 | 11 | 3 | 96 | 15 | 96 | 15 | 96 | 15 | 96 | 15 |
| 67 | Ranunculus acris | RanAcr | 76 | 3 | 46 | 3 | 46 | 3 | 309 | 13 | 309 | 15 | 301 | 15 | 299 | 15 |
| 68 | Ranunculus repens | RanRep | 40 | 3 | 25 | 3 | 25 | 3 | 170 | 15 | 170 | 15 | 165 | 15 | 165 | 15 |
| 69 | Rumex acetosa | RumAce | 126 | 3 | 93 | 3 | 92 | 3 | 608 | 9 | 608 | 11 | 607 | 11 | 605 | 11 |
| 70 | Rumex crispus | RumCri | 4 | 3 | 3 | 3 | 3 | 3 | 18 | 11 | 18 | 13 | 14 | 11 | 14 | 9 |
| 71 | Rumex obtusifolius | RumObt | 16 | 3 | 10 | 3 | 11 | 3 | 79 | 15 | 79 | 15 | 75 | 15 | 75 | 14 |
| 72 | Rumex thyrsiflorus | RumThy | 12 | 3 | 8 | 3 | 8 | 3 | 56 | 13 | 56 | 11 | 56 | 13 | 56 | 13 |
| 73 | Salvia pratensis | SalPra | 26 | 3 | 9 | 1 | 9 | 1 | 77 | 9 | 77 | 11 | 75 | 11 | 75 | 11 |
| 74 | Sanguisorba minor | SanMin | 8 | 3 | 2 | 2 | 2 | 3 | 22 | 14 | 22 | 14 | 22 | 10 | 22 | 12 |
| 75 | Saponaria officinalis | SapOff | 6 | 3 | 4 | 3 | 4 | 3 | 26 | 15 | 26 | 15 | 26 | 15 | 26 | 15 |
| 76 | Scabiosa columbaria | ScaCol | 18 | 3 | 6 | 3 | 6 | 3 | 56 | 15 | 56 | 15 | 56 | 15 | 56 | 15 |
| 77 | Scirpus sylvaticus | SciSyl | 52 | 3 | 13 | 2 | 12 | 1 | 117 | 11 | 117 | 9 | 116 | 11 | 117 | 11 |
| 78 | Sedum maximum | SedMax | 12 | 3 | 2 | 3 | 2 | 3 | 27 | 15 | 27 | 15 | 26 | 15 | 26 | 15 |
| 79 | Senecio jacobaea | SenJac | 56 | 3 | 36 | 3 | 37 | 3 | 263 | 15 | 263 | 15 | 260 | 15 | 260 | 15 |
| 80 | Silaum silaus | SilSil | 2 | 3 | 2 | 3 | 2 | 3 | 12 | 10 | 12 | 8 | 12 | 8 | 12 | 6 |
| 81 | Silene dioica | SilDio | 4 | 3 | 2 | 3 | 2 | 3 | 20 | 13 | 20 | 15 | 20 | 15 | 18 | 15 |
| 82 | Silene flos-cuculi | SilFlo | 64 | 3 | 40 | 3 | 40 | 3 | 293 | 9 | 293 | 14 | 293 | 15 | 293 | 15 |
| 83 | Silene latifolia | SilLat | 66 | 3 | 26 | 2 | 26 | 2 | 232 | 14 | 232 | 14 | 222 | 14 | 222 | 14 |
| 84 | Silene otites | SilOti | 30 | 3 | 9 | 1 | 9 | 1 | 77 | 7 | 77 | 7 | 77 | 7 | 77 | 7 |
| 85 | Silene vulgaris | SilVul | 4 | 3 | 2 | 2 | 2 | 1 | 16 | 11 | 16 | 11 | 16 | 11 | 16 | 11 |
| 86 | Symphytum officinale | SymOff | 6 | 3 | 2 | 3 | 2 | 3 | 19 | 15 | 19 | 15 | 19 | 15 | 19 | 15 |
| 87 | Thymus pulegioides | ThyPul | 12 | 3 | 3 | 3 | 3 | 3 | 38 | 11 | 38 | 13 | 38 | 13 | 38 | 13 |
| 88 | Tragopogon pratensis | TraPra | 6 | 3 | 2 | 1 | 2 | 1 | 21 | 9 | 21 | 9 | 20 | 10 | 20 | 10 |
| 89 | Trisetum flavescens | TriFla | 50 | 3 | 29 | 3 | 29 | 3 | 209 | 11 | 209 | 15 | 209 | 15 | 209 | 15 |
| 90 | Valeriana officinalis | ValOff | 18 | 3 | 8 | 3 | 8 | 3 | 60 | 15 | 60 | 15 | 60 | 13 | 60 | 15 |
| 91 | Veronica teucrium | VerTeu | 4 | 3 | 2 | 2 | 2 | 2 | 11 | 11 | 11 | 13 | 11 | 13 | 11 | 13 |
| 92 | Vicia cracca | VicCra | 18 | 3 | 2 | 3 | 2 | 3 | 28 | 15 | 28 | 13 | 28 | 15 | 27 | 15 |
| 93 | Vicia sepium | VicSep | 4 | 3 | 4 | 3 | 4 | 3 | 22 | 15 | 22 | 15 | 22 | 15 | 22 | 15 |

**Table S3:** Species performance variables in the field and common garden experiment (CG). The following performance variables of all 93 species (species abbreviations are given in Table S1) are shown: Survival (percentage of number individuals that survived till end of experiment in relation to number of individuals planted at the start of the experiment), biomass at end of experiment was log-transformed, mean values of RGR of height, RGR of plant projection area, RGR of leaf length and RGR of number of leaves is the species RGR mean across all 6 time intervals in field and accordingly 5 in common garden and of all individuals of one species [cm cm ^-1^ week^-1^] in field and common garden. Moreover, species scores of first (PC1) and second (PC2) axis are given for the two PCAs with all performance variables of field and common garden experiment respectively.

|  | Survival | | Biomass | | RGR height | | RGR plant proj. area | | RGR leaf length | | RGR number of leaves | | PC1 scores | | PC2 scores | |
| --- | --- | --- | --- | --- | --- | --- | --- | --- | --- | --- | --- | --- | --- | --- | --- | --- |
| Spec ID | Field | CG | Field | CG | Field | CG | Field | CG | Field | CG | Field | CG | Field | CG | Field | CG |
| AchMil | 78.26 | 100.00 | 0.057 | 1.757 | 0.005 | 0.136 | 0.033 | 0.129 | 0.015 | 0.044 | 0.012 | 0.146 | -0.461 | -0.390 | 0.259 | 0.000 |
| AgrEup | 50.00 | 100.00 | -0.611 | 1.178 | 0.011 | 0.086 | 0.017 | 0.154 | 0.013 | 0.060 | 0.011 | 0.055 | -0.198 | 0.231 | -0.275 | -0.215 |
| AllVin | 18.75 | 100.00 | -0.335 | 0.505 | 0.054 | 0.061 | -0.008 | 0.113 | -0.001 | 0.013 | -0.023 | -0.018 | 0.074 | 0.886 | -1.023 | 0.494 |
| AloPra | 16.67 | 100.00 | -0.484 | 2.030 | 0.005 | 0.062 | 0.004 | 0.150 | 0.012 | 0.073 | -0.018 | 0.087 | 0.170 | -0.150 | -0.661 | -0.105 |
| AntOdo | 82.14 | 100.00 | 0.124 | 1.819 | -0.011 | 0.001 | 0.016 | 0.123 | 0.017 | 0.061 | 0.017 | 0.106 | -0.342 | 0.027 | 0.545 | 0.461 |
| ArrEla | 32.61 | 100.00 | 0.611 | 2.112 | -0.014 | 0.068 | 0.018 | 0.117 | -0.003 | 0.046 | 0.011 | 0.102 | -0.163 | -0.191 | 0.560 | 0.357 |
| ArtVul | 41.67 | 100.00 | 1.266 | 2.430 | -0.007 | 0.147 | 0.007 | 0.199 | -0.024 | 0.037 | 0.033 | 0.204 | -0.287 | -1.085 | 1.261 | -0.331 |
| AstGly | 33.33 | 100.00 | 1.131 | 1.532 | 0.038 | 0.038 | 0.104 | 0.256 | 0.016 | 0.070 | 0.031 | 0.128 | -1.347 | -0.356 | 0.114 | -0.625 |
| BetOff | 42.86 | 100.00 | -0.484 | 1.210 | 0.004 | 0.141 | 0.030 | 0.180 | 0.005 | 0.068 | -0.001 | 0.114 | -0.105 | -0.176 | -0.250 | -0.651 |
| BraPin | 66.67 | 100.00 | -0.174 | 1.770 | -0.003 | 0.042 | 0.002 | 0.125 | 0.001 | 0.011 | -0.001 | 0.125 | 0.029 | -0.159 | 0.218 | 0.750 |
| BriMed | 61.67 | 100.00 | 0.037 | 1.783 | -0.002 | 0.041 | 0.027 | 0.170 | 0.013 | 0.079 | 0.011 | 0.134 | -0.336 | -0.246 | 0.210 | -0.160 |
| BroEre | 66.67 | 100.00 | 0.189 | 1.927 | -0.009 | 0.020 | 0.026 | 0.130 | 0.012 | 0.040 | 0.013 | 0.106 | -0.338 | -0.088 | 0.429 | 0.541 |
| CamRot | 75.00 | 100.00 | -0.838 | 1.042 | 0.003 | 0.109 | -0.028 | 0.168 | -0.009 | 0.010 | -0.043 | 0.106 | 0.665 | -0.047 | -0.358 | 0.103 |
| CarFla | 25.00 | 66.67 | -0.735 | 1.118 | 0.015 | 0.052 | 0.045 | 0.145 | 0.023 | 0.071 | 0.005 | 0.093 | -0.344 | 0.435 | -0.741 | -0.263 |
| CenJac | 59.52 | 100.00 | 0.336 | 2.019 | -0.003 | 0.151 | 0.034 | 0.193 | 0.012 | 0.027 | 0.009 | 0.169 | -0.411 | -0.792 | 0.329 | -0.275 |
| CenNig | 50.00 | 100.00 | 0.272 | 1.862 | -0.012 | 0.130 | 0.029 | 0.148 | 0.006 | 0.016 | 0.017 | 0.175 | -0.319 | -0.608 | 0.471 | 0.200 |
| CenSca | 50.00 | 66.67 | -0.704 | 1.523 | 0.002 | 0.072 | 0.016 | 0.124 | 0.006 | 0.053 | -0.006 | 0.020 | 0.055 | 0.571 | -0.309 | -0.099 |
| CicInt | 40.00 | 66.67 | 0.138 | 2.043 | 0.013 | 0.133 | 0.027 | 0.231 | 0.015 | 0.052 | 0.002 | 0.172 | -0.354 | -0.620 | -0.177 | -0.867 |
| CirAca | 50.00 | 100.00 | -0.239 | 1.453 | 0.022 | 0.103 | 0.023 | 0.160 | 0.019 | 0.070 | -0.009 | 0.073 | -0.254 | 0.020 | -0.500 | -0.389 |
| CynCri | 75.00 | 100.00 | 0.345 | 1.385 | 0.003 | 0.075 | 0.010 | 0.080 | -0.001 | 0.023 | 0.008 | 0.087 | -0.224 | 0.208 | 0.520 | 0.707 |
| DiaCar | 36.11 | 100.00 | -0.617 | 0.789 | -0.015 | 0.088 | 0.004 | 0.079 | 0.002 | 0.021 | -0.001 | 0.077 | 0.211 | 0.449 | -0.097 | 0.612 |
| DipFul | 21.43 | 100.00 | 0.620 | 2.119 | 0.013 | 0.105 | 0.003 | 0.130 | 0.006 | 0.047 | 0.001 | 0.054 | -0.191 | -0.105 | -0.008 | 0.047 |
| FesAru | 50.00 | 100.00 | -0.837 | 1.601 | -0.004 | 0.075 | 0.011 | 0.159 | 0.002 | 0.040 | 0.008 | 0.121 | 0.060 | -0.198 | -0.117 | 0.098 |
| FesGue | 100.00 | 100.00 | 0.172 | 1.559 | 0.011 | 0.030 | 0.009 | 0.063 | 0.010 | 0.027 | 0.014 | 0.149 | -0.389 | 0.025 | 0.493 | 1.048 |
| FesOvi | 71.43 | 100.00 | 0.172 | 1.757 | 0.004 | 0.022 | 0.008 | 0.053 | 0.008 | 0.012 | 0.011 | 0.153 | -0.257 | -0.036 | 0.349 | 1.309 |
| FesPra | 77.27 | 100.00 | 0.395 | 2.076 | 0.016 | 0.067 | 0.045 | 0.146 | 0.028 | 0.034 | 0.010 | 0.120 | -0.735 | -0.336 | 0.114 | 0.298 |
| FesRub | 78.57 | 100.00 | -0.394 | 1.646 | -0.023 | 0.034 | -0.010 | 0.135 | -0.011 | 0.037 | 0.003 | 0.144 | 0.276 | -0.172 | 0.596 | 0.488 |
| FilUlm | 60.00 | 100.00 | -0.944 | 1.541 | 0.031 | 0.116 | 0.045 | 0.148 | 0.041 | 0.068 | 0.005 | 0.046 | -0.584 | 0.097 | -0.931 | -0.375 |
| GalMol | 16.67 | 100.00 | -0.206 | 2.123 | 0.036 | 0.124 | 0.059 | 0.177 | 0.040 | 0.018 | 0.020 | 0.268 | -0.859 | -1.141 | -0.841 | 0.136 |
| GalPum | 50.00 | 100.00 | 0.465 | 2.102 | 0.023 | 0.094 | 0.064 | 0.194 | 0.003 | 0.006 | 0.051 | 0.277 | -0.978 | -1.166 | 0.510 | 0.288 |
| GerPra | 100.00 | 100.00 | -0.431 | 1.729 | 0.030 | 0.114 | 0.007 | 0.171 | 0.017 | 0.073 | -0.009 | 0.098 | -0.243 | -0.231 | -0.261 | -0.492 |
| GerPyr | 50.00 | 33.33 | -0.385 | 1.277 | -0.056 | 0.089 | -0.071 | 0.143 | -0.124 | 0.041 | -0.013 | 0.061 | 1.780 | 0.643 | 1.792 | -0.360 |
| GerSyl | 100.00 | 100.00 | -0.567 | 1.778 | 0.013 | 0.109 | 0.035 | 0.183 | 0.021 | 0.078 | -0.006 | 0.101 | -0.351 | -0.278 | -0.187 | -0.584 |
| GeuRiv | 66.67 | 100.00 | -0.430 | 1.505 | 0.005 | 0.070 | 0.027 | 0.121 | 0.010 | 0.055 | 0.014 | 0.071 | -0.291 | 0.157 | 0.051 | 0.160 |
| GeuUrb | 83.33 | 100.00 | -0.555 | 2.017 | 0.009 | 0.090 | 0.007 | 0.102 | 0.011 | 0.039 | -0.013 | 0.083 | -0.004 | -0.090 | -0.189 | 0.387 |
| HelNum | 33.33 | 33.33 | -1.469 | 0.976 | 0.015 | 0.045 | -0.030 | 0.176 | -0.021 | 0.037 | -0.033 | 0.136 | 0.829 | 0.454 | -0.838 | -0.278 |
| HelPub | 50.00 | 100.00 | -0.171 | 1.992 | -0.009 | 0.008 | 0.021 | 0.114 | 0.015 | 0.037 | 0.001 | 0.109 | -0.146 | -0.063 | -0.004 | 0.733 |
| HiePil | 50.00 | 66.67 | -0.440 | 0.639 | -0.009 | 0.008 | 0.000 | 0.192 | -0.004 | 0.002 | -0.007 | 0.201 | 0.226 | 0.083 | 0.029 | 0.357 |
| HolLan | 82.93 | 100.00 | 0.365 | 2.332 | -0.013 | 0.080 | 0.016 | 0.149 | 0.001 | 0.071 | 0.011 | 0.106 | -0.234 | -0.370 | 0.769 | -0.118 |
| HypPer | 64.29 | 100.00 | -0.271 | 1.970 | 0.010 | 0.104 | -0.003 | 0.122 | 0.002 | 0.024 | 0.002 | 0.142 | -0.020 | -0.398 | 0.028 | 0.386 |
| HypRad | 100.00 | 33.33 | -0.221 | 0.862 | 0.019 | 0.121 | 0.035 | 0.146 | 0.016 | 0.042 | -0.007 | 0.061 | -0.405 | 0.735 | -0.067 | -0.569 |
| KnaArv | 50.00 | 100.00 | -0.271 | 2.054 | -0.006 | 0.116 | -0.012 | 0.153 | 0.000 | 0.051 | -0.002 | 0.091 | 0.190 | -0.305 | 0.100 | -0.168 |
| KoePyr | 100.00 | 100.00 | -0.498 | 1.668 | 0.015 | 0.062 | 0.010 | 0.086 | 0.011 | 0.025 | -0.014 | 0.112 | -0.085 | 0.010 | -0.123 | 0.743 |
| LatPra | 30.00 | 100.00 | -0.573 | 1.775 | -0.018 | 0.066 | 0.015 | 0.137 | -0.014 | 0.024 | -0.049 | 0.125 | 0.617 | -0.220 | -0.423 | 0.442 |
| LeoAut | 50.00 | 33.33 | -0.199 | 1.265 | 0.005 | 0.142 | 0.007 | 0.150 | 0.006 | 0.044 | -0.004 | 0.068 | -0.026 | 0.508 | -0.102 | -0.672 |
| LeoHis | 50.00 | 66.67 | -0.232 | 1.231 | -0.002 | 0.083 | 0.033 | 0.134 | 0.015 | 0.043 | 0.023 | 0.098 | -0.408 | 0.319 | 0.111 | -0.065 |
| LeuVul | 50.00 | 33.33 | -0.076 | 0.022 | -0.044 | 0.106 | -0.006 | 0.113 | -0.005 | 0.026 | -0.007 | 0.034 | 0.390 | 1.258 | 0.581 | -0.221 |
| LolPer | 42.86 | 100.00 | 0.126 | 1.878 | -0.008 | 0.079 | -0.008 | 0.089 | -0.001 | 0.013 | -0.007 | 0.150 | 0.159 | -0.270 | 0.189 | 0.822 |
| LotCor | 55.56 | 100.00 | 0.437 | 1.502 | 0.004 | 0.119 | 0.024 | 0.165 | 0.002 | 0.043 | 0.001 | 0.126 | -0.266 | -0.277 | 0.300 | -0.185 |
| LuzCam | 61.54 | 100.00 | -0.142 | 1.281 | -0.016 | 0.019 | 0.008 | 0.088 | 0.007 | 0.032 | -0.007 | 0.098 | 0.059 | 0.292 | 0.184 | 0.826 |
| MedFal | 50.00 | 100.00 | 0.010 | 2.038 | -0.027 | 0.090 | -0.009 | 0.214 | -0.009 | 0.005 | -0.018 | 0.206 | 0.404 | -0.907 | 0.361 | 0.119 |
| MedLup | 8.33 | 33.33 | -0.947 | -0.420 | -0.032 | 0.066 | -0.018 | 0.166 | -0.028 | -0.025 | -0.030 | 0.096 | 0.965 | 1.066 | -0.227 | 0.133 |
| MedXva | 83.33 | 100.00 | 0.589 | 2.629 | 0.011 | 0.076 | 0.041 | 0.202 | 0.001 | -0.015 | 0.022 | 0.225 | -0.634 | -1.168 | 0.686 | 0.508 |
| OriVul | 60.00 | 100.00 | -0.637 | 1.938 | 0.002 | 0.127 | -0.007 | 0.133 | -0.002 | 0.022 | -0.016 | 0.117 | 0.288 | -0.360 | -0.202 | 0.197 |
| PasSat | 26.92 | 33.33 | -0.354 | 1.201 | -0.004 | 0.115 | 0.004 | 0.153 | -0.013 | 0.092 | 0.006 | 0.089 | 0.179 | 0.537 | 0.058 | -1.003 |
| PhlPhl | 40.00 | 100.00 | -0.198 | 1.441 | 0.005 | 0.029 | 0.002 | 0.121 | -0.004 | 0.045 | -0.031 | 0.102 | 0.293 | 0.123 | -0.341 | 0.460 |
| PicHie | 35.00 | 66.67 | -0.233 | 1.985 | 0.014 | 0.158 | 0.010 | 0.190 | 0.002 | 0.058 | 0.004 | 0.138 | -0.091 | -0.400 | -0.198 | -0.815 |
| PimMaj | 66.67 | 66.67 | -0.900 | 1.205 | 0.015 | 0.041 | 0.013 | 0.148 | 0.016 | 0.064 | -0.011 | 0.047 | -0.030 | 0.590 | -0.556 | -0.204 |
| PimSax | 25.00 | 66.67 | -0.419 | 1.067 | 0.053 | 0.193 | 0.049 | 0.187 | 0.036 | 0.079 | 0.005 | 0.068 | -0.727 | 0.189 | -1.160 | -1.294 |
| PlaLan | 70.00 | 66.67 | 0.441 | 1.386 | -0.016 | 0.119 | 0.014 | 0.094 | 0.000 | 0.038 | 0.016 | 0.097 | -0.216 | 0.294 | 0.807 | 0.079 |
| PlaMed | 66.67 | 100.00 | -0.206 | 1.235 | 0.017 | 0.129 | 0.042 | 0.097 | 0.025 | 0.036 | 0.005 | 0.065 | -0.529 | 0.218 | -0.259 | 0.190 |
| PoaAng | 25.00 | 100.00 | -1.602 | 1.083 | -0.057 | -0.018 | 0.026 | 0.124 | -0.005 | 0.068 | 0.032 | 0.031 | 0.314 | 0.646 | 0.256 | 0.360 |
| PoaPra | 75.00 | 100.00 | -0.784 | 1.408 | -0.026 | -0.010 | -0.018 | 0.141 | -0.003 | 0.073 | -0.026 | 0.059 | 0.589 | 0.354 | 0.077 | 0.210 |
| PotArg | 39.19 | 100.00 | -0.392 | 1.499 | 0.001 | 0.102 | 0.004 | 0.161 | 0.003 | 0.022 | -0.001 | 0.156 | 0.067 | -0.372 | -0.150 | 0.146 |
| PruVul | 67.86 | 100.00 | 0.484 | 1.315 | 0.002 | 0.114 | 0.025 | 0.158 | 0.006 | 0.030 | 0.007 | 0.121 | -0.370 | -0.173 | 0.435 | -0.017 |
| PseSpi | 50.00 | 100.00 | -0.385 | 1.268 | -0.002 | 0.076 | 0.007 | 0.140 | -0.003 | 0.032 | 0.001 | 0.093 | 0.071 | 0.076 | 0.041 | 0.229 |
| RanAcr | 60.53 | 100.00 | -0.275 | 1.304 | 0.003 | 0.072 | -0.002 | 0.171 | 0.013 | 0.066 | -0.013 | 0.049 | 0.056 | 0.198 | -0.185 | -0.297 |
| RanRep | 65.79 | 100.00 | -0.014 | 1.763 | -0.011 | 0.081 | 0.014 | 0.254 | -0.010 | 0.056 | -0.003 | 0.139 | 0.049 | -0.577 | 0.428 | -0.659 |
| RumAce | 76.23 | 100.00 | -0.622 | 1.271 | -0.038 | -0.022 | -0.009 | 0.194 | -0.002 | 0.073 | -0.025 | 0.108 | 0.542 | 0.096 | 0.276 | -0.033 |
| RumCri | 75.00 | 100.00 | 0.289 | 1.481 | -0.012 | 0.134 | 0.030 | 0.113 | -0.024 | 0.049 | -0.003 | 0.120 | -0.018 | -0.133 | 0.761 | 0.012 |
| RumObt | 62.50 | 100.00 | -1.270 | 2.170 | -0.036 | 0.136 | -0.054 | 0.160 | -0.022 | 0.045 | -0.027 | 0.045 | 1.120 | -0.227 | 0.110 | -0.273 |
| RumThy | 66.67 | 100.00 | -0.485 | 1.854 | -0.007 | 0.209 | -0.014 | 0.231 | -0.007 | 0.075 | -0.030 | 0.122 | 0.468 | -0.702 | -0.073 | -1.291 |
| SalPra | 34.62 | 33.33 | -0.347 | 1.229 | -0.013 | 0.032 | 0.011 | 0.101 | 0.004 | 0.052 | 0.002 | 0.035 | 0.073 | 0.992 | -0.002 | 0.045 |
| SanMin | 25.00 | 66.67 | -0.232 | 1.210 | -0.001 | 0.091 | -0.013 | 0.106 | 0.004 | 0.007 | -0.016 | 0.095 | 0.289 | 0.363 | -0.311 | 0.408 |
| SapOff | 66.67 | 100.00 | -1.027 | 1.901 | -0.006 | 0.082 | -0.015 | 0.158 | -0.012 | 0.040 | -0.020 | 0.142 | 0.541 | -0.406 | -0.164 | 0.103 |
| ScaCol | 33.33 | 100.00 | 0.113 | 1.578 | 0.027 | 0.135 | 0.023 | 0.183 | 0.016 | 0.062 | 0.003 | 0.097 | -0.389 | -0.252 | -0.383 | -0.578 |
| SciSyl | 25.00 | 66.67 | 1.055 | 0.086 | 0.016 | 0.070 | 0.062 | 0.197 | 0.036 | 0.091 | 0.011 | 0.030 | -0.940 | 0.927 | -0.098 | -1.003 |
| SedMax | 16.67 | 100.00 | -2.222 | 0.192 | -0.042 | 0.049 | -0.034 | 0.170 | -0.018 | 0.037 | -0.052 | 0.033 | 1.443 | 0.701 | -0.893 | -0.020 |
| SenJac | 64.29 | 100.00 | 0.154 | 2.223 | 0.029 | 0.158 | 0.024 | 0.182 | 0.013 | 0.063 | 0.011 | 0.139 | -0.505 | -0.697 | -0.043 | -0.596 |
| SilSil | 100.00 | 100.00 | -0.985 | 1.213 | 0.010 | 0.086 | 0.015 | 0.206 | 0.008 | 0.066 | -0.006 | 0.097 | -0.039 | -0.077 | -0.169 | -0.554 |
| SilDio | 50.00 | 100.00 | -0.308 | 1.465 | -0.034 | 0.077 | 0.002 | 0.101 | 0.005 | 0.046 | -0.005 | 0.024 | 0.244 | 0.388 | 0.278 | 0.289 |
| SilFlo | 62.50 | 100.00 | -0.184 | 1.442 | -0.050 | -0.008 | 0.003 | 0.086 | 0.000 | 0.015 | -0.002 | 0.049 | 0.293 | 0.466 | 0.679 | 1.092 |
| SilLat | 39.39 | 66.67 | -0.020 | 1.656 | -0.021 | 0.100 | -0.009 | 0.183 | -0.016 | 0.015 | -0.015 | 0.124 | 0.430 | -0.134 | 0.315 | -0.136 |
| SilOti | 30.00 | 33.33 | -0.648 | 0.460 | 0.006 | 0.129 | 0.011 | 0.162 | 0.010 | 0.031 | -0.009 | 0.062 | 0.071 | 0.818 | -0.544 | -0.624 |
| SilVul | 50.00 | 66.67 | -0.698 | 0.558 | 0.015 | 0.075 | -0.019 | 0.165 | -0.019 | 0.001 | -0.057 | 0.092 | 0.732 | 0.491 | -0.670 | 0.113 |
| SymOff | 33.33 | 100.00 | -0.425 | 2.332 | -0.039 | 0.071 | -0.040 | 0.200 | -0.025 | 0.070 | -0.012 | 0.119 | 0.847 | -0.539 | 0.445 | -0.375 |
| ThyPul | 25.00 | 100.00 | -1.232 | 1.192 | -0.001 | 0.051 | -0.012 | 0.149 | -0.007 | 0.040 | -0.020 | 0.155 | 0.575 | -0.110 | -0.670 | 0.269 |
| TraPra | 33.33 | 33.33 | -0.378 | 0.385 | -0.009 | 0.045 | 0.002 | 0.141 | 0.007 | 0.065 | -0.005 | 0.084 | 0.142 | 1.000 | -0.166 | -0.414 |
| TriFla | 58.00 | 100.00 | -0.114 | 1.313 | -0.008 | 0.067 | 0.021 | 0.162 | 0.003 | 0.049 | 0.011 | 0.092 | -0.163 | 0.041 | 0.293 | -0.023 |
| ValOff | 44.44 | 100.00 | -0.526 | 1.901 | 0.014 | 0.136 | -0.002 | 0.123 | 0.011 | 0.042 | -0.014 | 0.106 | 0.093 | -0.273 | -0.512 | 0.020 |
| VerTeu | 50.00 | 66.67 | -0.754 | 1.406 | 0.054 | 0.062 | 0.051 | 0.165 | 0.007 | 0.027 | -0.008 | 0.096 | -0.429 | 0.199 | -0.970 | 0.000 |
| VicCra | 11.11 | 100.00 | -1.328 | 1.142 | -0.008 | 0.055 | -0.030 | 0.122 | -0.002 | 0.046 | -0.010 | 0.121 | 0.664 | 0.112 | -0.646 | 0.324 |
| VicSep | 100.00 | 100.00 | 0.260 | 1.966 | 0.004 | 0.056 | 0.041 | 0.159 | 0.008 | 0.041 | 0.020 | 0.099 | -0.601 | -0.218 | 0.655 | 0.177 |

**Table S4:** Spearman correlation coefficients for performance variables (biomass, survival, RGR of height, plant projection area, leaf length and number of leaves) between observations on mean species values in the field and the common garden experiment.

|  | **Spearman's rank correlation rho** | **p-value** |
| --- | --- | --- |
| **Biomass** | 0.4225 | 0.0000 |
| **Survival** | 0.3149 | 0.0021 |
| **RGR height** | 0.2765 | 0.0075 |
| **RGR plant projection area** | 0.1179 | 0.2597 |
| **RGR leaf length** | 0.3166 | 0.0021 |
| **RGR number of leaves** | 0.2145 | 0.0392 |


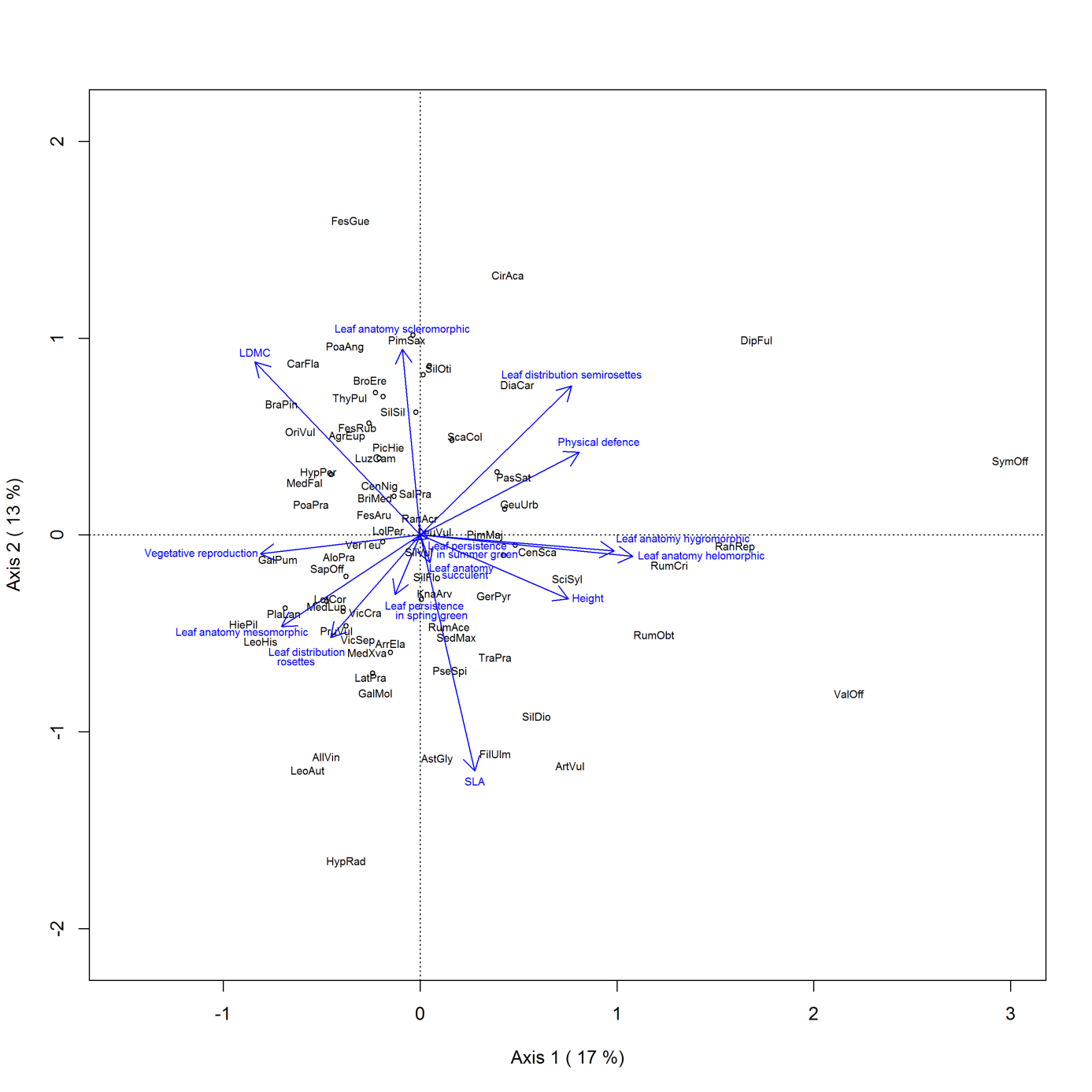


**Figure S1:** Principal component analysis (PCA) based on 14 traits (SLA, LDMC, height, leaf anatomy (succulent, scleromorphic, mesomorphic, hygromorphic and helomorphic), leaf persistence (in spring green and in summer green), leaf distribution (rosettes and semirosettes), physical defense and vegetative reproduction) and all 93 species (for abbrevations of species names see supporting information Table S2).
